# Characterization of Mass Transfer within the Crystal-Solution Boundary Layer of l-Alanine {120} Faces Using Laser Interferometry during Growth and Dissolution

**DOI:** 10.1021/acs.cgd.2c01541

**Published:** 2023-03-16

**Authors:** Steven T. Nicholson, Kevin J. Roberts, Toshiko Izumi, Xiaojun Lai

**Affiliations:** † EPSRC Centre for Doctoral Training in Complex Particulate Products and Processes, School of Chemical and Process Engineering, University of Leeds, Woodhouse Lane, Leeds, LS2 9JT, United Kingdom; ‡ Pfizer R&D U.K., Ramsgate Road, Sandwich, Kent CT13 9NJ, United Kingdom

## Abstract

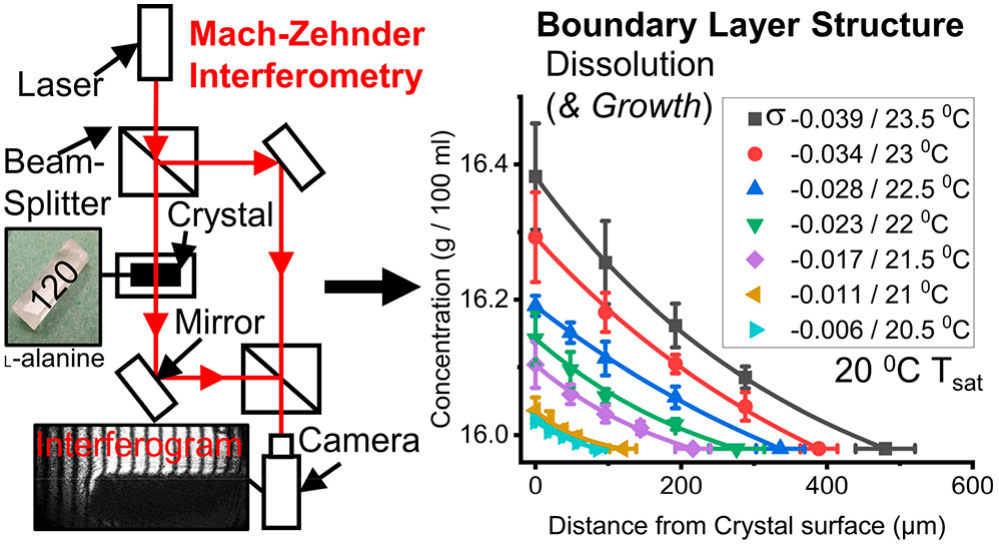

Crystallization and dissolution are important processes to consider in drug development as well as many other industrial processes. Many current growth and dissolution models are based on bulk solution properties and do not implicitly consider concentration variation close to the crystal surface–solution interface and how this is mediated by solute diffusive mass transfer. Solution boundary layer thickness and concentration distribution, for the {120} crystal habit face of single crystals of l-alanine in saturated aqueous solutions during both growth and dissolution processes, is measured as a function of super/undersaturation using a Mach–Zehnder optical interferometer system. Further analysis allows determination of the diffusion coefficient and mass flux within the boundary layer as well as whether the processes are controlled by mass transfer or crystal interfacial kinetics. The measurement of this study revealed that the {120} face was not saturated at its surface during growth or dissolution meaning both processes were somewhat limited by their crystal interfacial kinetics. Growth was limited by crystal interfacial kinetics at all supersaturations to the same degree, whereas dissolution had a mixed dependency on crystal interfacial kinetics and mass transfer at lower undersaturations becoming more limited by mass transfer at higher undersaturations. Boundary layer thickness increased with super/undersaturation but to a lesser degree than the increase in the concentration difference between the crystal surface and bulk solution leading to a higher mass flux of solute molecules through the boundary layer. At the same relative super/undersaturation mass flux of solute molecules was faster during dissolution which was concurrent with its increased surface to bulk solution concentration difference and boundary layer thickness.

## Abstract 2

Boundary layer interferometric analysis of l-alanine reveals rate-limiting processes for growth and dissolution.

## 1 Introduction

There are an increasing number of complex active pharmaceutical ingredients (APIs) being developed which fall into the biopharmaceutical classification system (BCS) classes II/IV and, hence, have poor solubility in the gastrointestinal (GI) tract exhibiting slow dissolution rate behavior with concomitantly low bioavailability.^1−4^ The design of poorly soluble drug compounds to overcome these issues with bioavailability remains a persistent challenge due to an insufficient understanding of the optimal dissolution characteristics from first principles. Dissolution is also an important process in the wider chemical industry, such as the nuclear industry, and similar areas such as physics, biology, and environmental science.^5,6^ Dissolution and crystal growth can be considered to be a series combination of mass transfer (diffusion down a concentration gradient away from/toward the crystal surface into/from the bulk solution) and crystal interfacial kinetics (the mechanism by which a molecule detaches or attaches itself from/to a crystal surface).^7,8^ However, current dissolution models are still based on the Noyes-Whitney equation:
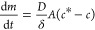
1where the change in mass over time (d*m*/d*t*, kg s^–1^) is described by the diffusion coefficient (*D*, m^2^ s^–1^), boundary layer (BL) thickness (δ, m), surface area (*A*, m^2^), and the difference in the equilibrium solution concentration and the solute concentration in the bulk solution (c^*^ – *c*, kg m^–3^).^9−11^ This model is based on bulk properties of the crystal and assumes diffusional processes govern dissolution. It does not take into account the actual solution concentration/undersaturation at crystal dissolving surfaces. The Noyes-Whitney equation assumes the solution around the crystal surface is saturated: the rate of detachment of molecules from the surface does not contribute to the dissolution rate. If this was the case then all the crystal faces of a dissolving crystal would dissolve at the same rate, but this is not what is observed.^12,13^ Subsequently, it does not accurately describe diffusion from the crystal surface into the bulk solution from these crystal surfaces as it must be the case that the crystal interfacial kinetics is slow enough to impact the dissolution rate and the surface chemistry (the way in which the molecule is orientated on the crystal surface and how the functional groups interact with the solution) must play an important role in dissolution, the subsequent surface concentration and overall boundary layer structure.

**Figure 1:**
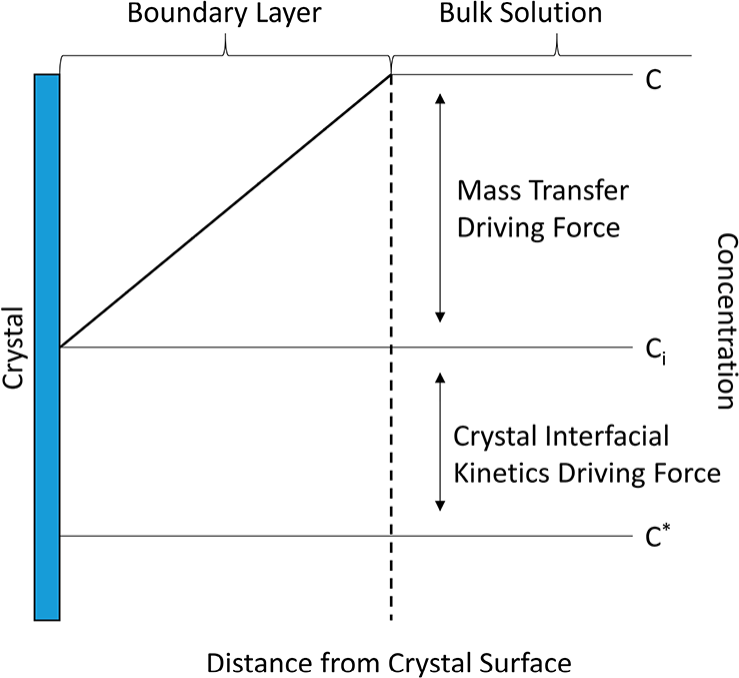
Schematic example of the overall diffusion boundary layer structure during crystal growth showing the concentration in the bulk solution, *c*, the subsequent concentration distribution in the boundary layer going from the bulk solution to the crystal surface, the boundary layer thickness (dashed line), the concentration at the interface, *c*_i_, and the equilibrium solute concentration, c^*^. The relative driving forces for crystal growth are depicted: the mass transfer driving force and the crystal interfacial kinetics driving force.^7,28,29^

Crystallization is a comparatively well-studied phenomenon and is affected by solid form parameters such as polymorphism, crystal morphology, and size.^7,14^ It is widely used in the chemical industry (for the purification and isolation of APIs as well as other chemical products and can impact product quality, flowability, and bioavailability) as well as in other closely related areas.^15−18^ Dissolution is often considerably faster than growth so it is clear that the two processes can differ on a molecular level.^7,19^ This can be caused by competing processes such as 2-D/3-D nucleation during growth, the increased number of active sites from edge dislocations and point defects during dissolution, impurities in the crystal lattice slowing growth but speeding up dissolution, the activities of etch pits, etc.^20−24^ It is well established that crystal faces of a particular crystal can grow at different rates.^25−27^ This implies that crystal interfacial kinetics has a large impact on the growth rates of different crystal faces. A quantitative measure of crystal interfacial kinetics or mass transfer control can be determined through calculation of an effectiveness factor.^7,28,29^ Similarly to dissolution, crystal growth models are often based on bulk solution properties of crystals, such as the growth model for 2-D nucleation on a crystal surface.^7,30^ It still remains unclear how the surface concentration is related to the surface chemistry of *hkl* faces during growth, and the crystal interfacial kinetics coefficient (*k*_r_, m s^–1^) in diffusion-reaction based models remains undefined:
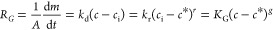
2where *R*_G_ (kg m^–2^ s^–1^) is the growth rate, *k*_d_ (m s^–1^) is the coefficient of mass transfer, *K*_G_ (m s^–1^) is the overall crystal growth coefficient, *c*_i_ (kg m^–3^) is the surface concentration, and *r/g* are the orders of the crystal interfacial kinetics and overall growth processes, respectively.^7,31,32^Equation 2 can be visualized in Figure 1 which shows an overall schematic of the diffusion boundary layer during crystal growth. During growth, a concentration gradient is created between the concentration in the bulk solution, *c*, and concertation at the crystal surface, *c*_i_, creating a concentration distribution within the resultant boundary layer. The driving force for crystal growth can be controlled either by the mass transfer through the boundary layer, the crystal interfacial kinetics or both depending on the relative differences between *c*, *c*_i_, and c^*^.^7,28,29^ There is a need to elucidate how the boundary layer structure during crystal growth relates to the surface chemistry of crystal faces as well as understanding the differences between growth and dissolution with regard to their boundary layer structure through observation of the crystal surface-solution interface on different crystal surfaces.

Many techniques have been used for solution and crystal observation during growth and dissolution including microscopy,^20,22,23,33−35^ SICM,^12^ AFM,^13^ and the Schlieren technique.^36^ Previous work has also used Michelson interferometry to study crystal growth through measurement of face-specific growth rates as a function of supersaturation and temperature,^37,38^ observing spiral growth steps,^22,23,33^ measuring boundary layer concentration,^39−42^ and studying the effects of flow on the growth rate.^23,34^ It has also been used sparingly for the study of dissolution.^20,23,43,44^ Mach–Zehnder interferometry has been used for the measurement of concentration around a growing crystal^22,24,36,45,46^ and has also been used in combination with Michelson interferometry to simultaneously measure crystal growth/dissolution rates and boundary layer concentrations.^20,24^ These interferometers have the advantage of a simple design, flexible setup, and ease of use while also being extremely accurate techniques.^47,48^ In order to characterize the boundary layer and solute concentration variation from the growth/dissolution interface as well as its thickness, diffusion coefficient, and mass flux, a Mach–Zehnder interferometer was used in this study. Although work has been done studying the surface concentration and boundary layer thickness, this work is scarce, and there remains a gap in knowledge regarding how the concentration distribution and mass flux within the boundary layer relates to the surface concentration and surface chemistry of the crystal.

**Figure 2:**
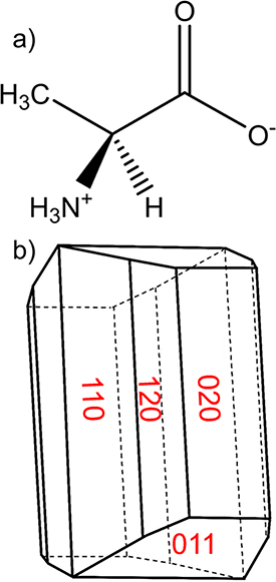
Molecular structure of l-alanine in its zwitterionic form (a) and the resultant sketch of its morphology (b).

Single crystals of l-alanine grown from aqueous solution provide a useful model system of study. Its molecular structure in its zwitterionic form and sketch of its morphology can be seen in Figure 2. This compound is one of the smallest amino acids and has a wide range of applications from the pharmaceutical industry to the food industry.^49^ Its aqueous solution characteristics are optimal for the growth of large single crystals and, previously, it has been grown to large sizes (cm^3^) for study as a nonlinear optical material.^50−52^ Such large crystals are essential for observation of the crystal–solution interface using interferometry. l-Alanine’s solubility in water has been measured previously revealing a shallow positive exponential trend with temperature while its metastable zone width (MSZW) is quite wide (∼12–20 °C).^53−60^ As the latter increases with increasing saturation temperature this indicates lower solution supersaturations and higher temperatures would provide optimal conditions for stable growth. Its crystal morphology displays a number of large faces including the {120}, {110}, {011}, and {020} faces.^51,61,62^ The {120} face was chosen for this study due to its large size and shape allowing the laser to be in contact with the solution around the crystal surface for a significant period of time. The molecule is zwitterionic and crystallizes in an orthorhombic crystal structure, space group *P*2_1_2_1_2_1_, in a tetra-molecular unit cell, with dimensions *a* = 6.032 Å, *b* = 12.343 Å, *c* = 5.784 Å, with a crystal chemistry encompassing a network of interconnected hydrogen bonds.^59,63^ Its lattice energy, morphology, and surface chemistry have been modeled previously.^49,64^

This paper reports a study of single crystals of l-alanine using Mach–Zehnder interferometry to characterize the stagnant solution boundary layer concentration distribution, thickness, diffusion coefficient, and mass flux during both growth and dissolution of the {120} face as a function of solution super/undersaturation revealing whether the processes were controlled by crystal interfacial kinetics or mass transfer. These measurements, together with determination of solution properties of the system (solubility, refractive index, and viscosity), were used to elucidate the differences in boundary layer structure for both the growth and dissolution cases, as a function of supersaturation and undersaturation respectively, taking into account face-specific growth and dissolution mechanisms. This paper also provides a workflow for boundary layer measurements based upon crystal face-specific values. Mapping of the concentration distribution within the boundary layer allowed the mass flux to be calculated providing the first step in the development of a new growth and dissolution model. The overall project aims to understand how the boundary layer structure relates to the surface chemistry of different crystal faces.

## 2 Materials and Methods

### 2.1 Materials

l-Alanine (C_3_H_7_NO_2_), molecular weight: 89.094, percent purity: 99%, was purchased from Fisher Scientific. Deionized water was used as the solvent for crystal growth and dissolution. Pure Beeswax was purchased from Fisher Scientific and used to secure the crystals to the cell.

### 2.2 Preparation of Single Crystals of l-Alanine

Aqueous solutions of l-alanine were prepared at room temperature and solvent evaporation was used to grow crystals with dimensions ∼5 × 3 × 2 mm^3^. Crystals were further grown to larger sizes for morphology determination using a specialized crystal growth rig (see Supporting Information S1 for the large single crystal growth method and Figure S1 for the crystal growth rig equipment).^65,66^ Seed crystals were suspended in a saturated solution at 40 °C and the temperature was lowered by 1 °C per day to achieve crystals with sizes ∼3 × 1 × 1 cm^3^.

### 2.3 Crystal and Solution Properties

The morphology was determined by measuring the interfacial angles of the large crystals grown using the crystal growth rig combined with unit cell parameters (see Supporting Information S2 for the morphology determination method).^63^ The LALNIN12 structure, downloaded from the crystallographic structural database (CSD) in Mercury, was used to calculate the lattice energy and attachment energies of l-alanine using Habit98 (see Supporting Information S3 for the crystal chemistry modeling method).^67−71^ The predicted morphology was then plotted in Mercury.

The MSZW was determined using the polythermal crystallization method in a Technobis Crystal 16 unit (see Supporting Information S4 and Figure S2 for the method for determination of the MSZW using polythermal crystallization). Four solution were used (0.177, 0.188, 0.198, and 0.208 g mL^–1^) at four cooling/heating rates to obtain the average temperatures of crystallization and dissolution. Extrapolation to a 0 °C cooling rate revealed the MSZW. Solubility was determined using the gravimetric method on saturated solutions prepared at 5 °C intervals between 10 and 50 °C, and the relative super/undersaturations used in this study were calculated using eq S1 (see Supporting Information S5 for the method to determine solubility). Viscosity was determined using an Anton Paar Physica MCR301 Rheometer on saturated solutions prepared at 5 °C intervals between 10 and 35 °C (see Supporting Information S6 for the method to determine viscosity). For each saturated solution the temperature was varied from 4 °C above the saturation temperature to 4 °C below the saturation temperature with measurements taken at 1 °C intervals for the viscosity.

### 2.4 Refractive Index

Refractive index was measured using an Abbe 60 Refractometer. Solutions were prepared by shaking an excess of solute in solvent at 5 °C intervals between 10 and 50 °C using an incubated shaker at 250 rpm. The resulting solutions were filtered and stirred above their saturation temperature before being pipetted onto the sample stage of the Refractometer. The temperature was altered from 4 °C above the saturation temperature to 4 °C below the saturation temperature taking refractive index measurements every 2 °C. The temperature of the stage was manipulated through the use of an external bath. A torch illuminated the sample within the stage and the adjustment wheels were used to focus and align the crosshairs in the upper window so the light/dark boundary lined up with the crosshairs. The refractive index measurement was then read from the lower window.

### 2.5 Interferometer Setup

**Figure 3:**
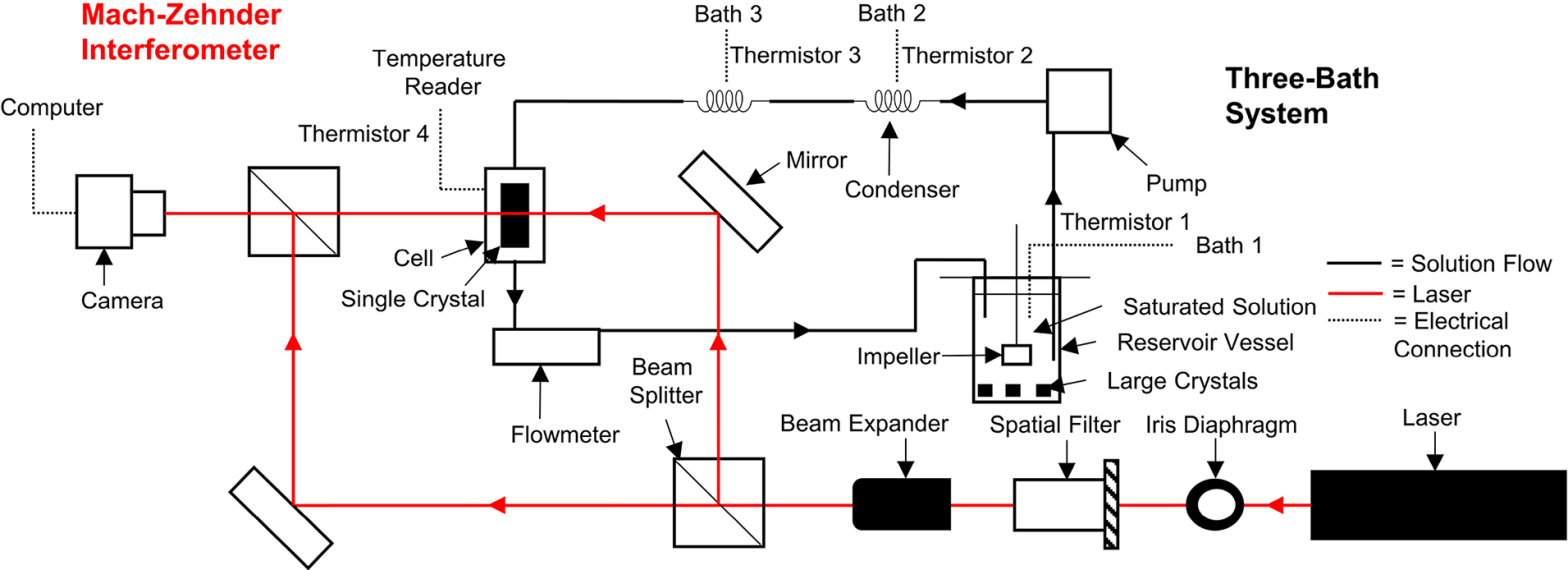
Schematic of the Mach–Zehnder interferometer and three-bath system set up, used to control the super/undersaturation of the system.

The Mach–Zehnder interferometer is shown in Figure 3 and consists of a He–Ne laser (λ_0_ = 632.8 nm, power = 2–3 mW); an iris diaphragm, which helps reduce the scatter of the laser improving the interferogram; a spatial filter, which helps improve the lateral uniformity of the laser also improving the interferogram quality; and a beam expander, which expands the beam to a diameter of ∼2 cm allowing for detection of a larger area. The laser beam is then split into two paths: the “reference” beam and “sample” beam. The sample beam transmits through a specially designed cell interacting with the solution around the growing or dissolving crystal. The reference beam is diverted around the cell. The beams converge creating interference which is detected by the camera. The design is based on multiple designs used previously.^20,22,24,36,45,46^

#### 2.5.1 In Situ Cell for Crystal Growth and Dissolution Experiments

**Figure 4:**
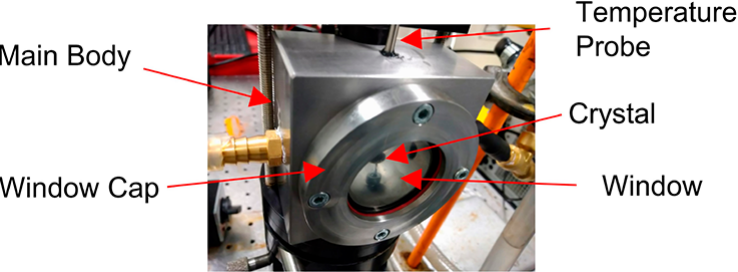
Image of the specially designed Mach–Zehnder cell manufactured in-house.

An in situ stainless steel cell, which was designed and manufactured in-house and consisted of two parts, the main body and the window caps, is shown in Figure 4. The crystal was mounted on a stand in the middle of the cell. The solution flowed from one side of the cell to the other. A temperature probe was situated at the top of the cell attached to a temperature reader. The window caps acted to attach the windows to the cell and create a seal. The crystal (size: ∼5 × 3 × 2 mm^3^, ∼1.1 mm {120} face length) was secured onto the stage using Beeswax and positioned so that the selected crystal habit face was aligned with the beam transmitting through the cell.

#### 2.5.2 Temperature Control System

The temperature control system is shown in Figure 3 (Three-Bath System) and worked to maintain the saturation temperature while altering the super/undersaturation in the cell to allow for growth or dissolution. A saturated solution was prepared at 20 °C and placed into the jacketed reservoir vessel overnight with large crystals present in the vessel. The solution was then pumped from the bottom of the reservoir vessel through a jacketed condenser coil where the temperature was held 3–4 °C above the saturation temperature to dissolve minute crystalline material that may interfere with the experiment. The solution then flowed into a second condenser coil which controlled the super/undersaturation of the solution by changing the temperature from 16.5 to 23.5 °C whereby the temperature was kept steady at 0.5 °C intervals (super/undersaturations: 0.041/-0.039, 0.035/-0.034, 0.029/-0.028, 0.023/-0.023, 0.017/-0.017, 0.012/-0.011, and 0.006/-0.006). The system was allowed to flow for 2–3 h, with bath 3 at 16.5 °C, prior to the experiment to allow for the correct temperatures to be attained and minute crystalline material to dissolve. Pumping was then briefly stopped and the in situ cell was placed into the flow system. Images were taken when the temperature reader attached to the cell displayed the same temperature as bath 3 for 15 min to allow for equilibration. The solution then flowed through a flowmeter which kept the flow rate at 0.5 cm^3^ s^–1^.

### 2.6 Mach–Zehnder Interferometry Measurement

**Figure 5:**
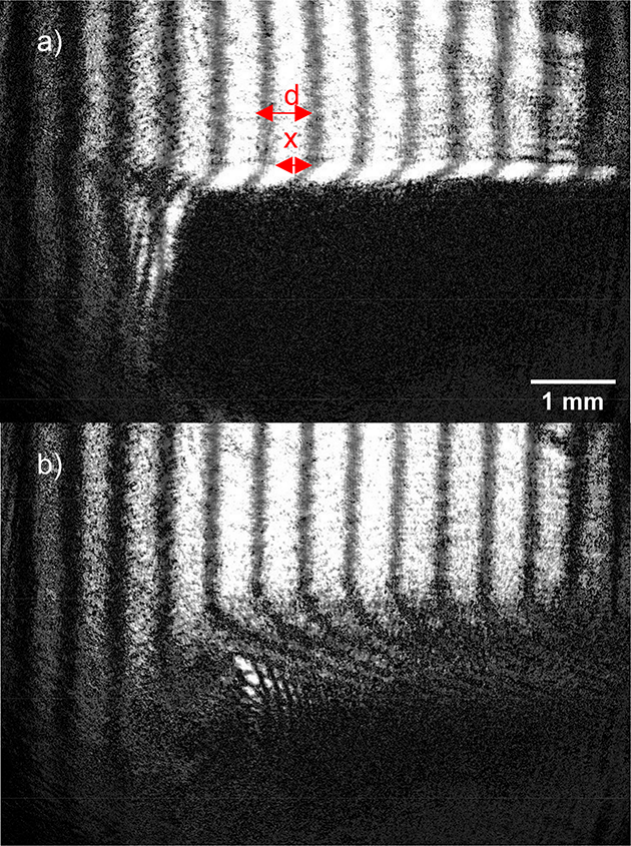
Example of interferograms taken during growth (a) and dissolution (b) with the interference fringes aligned to the {120} face.

Interference fringes arise due to the phenomenon of constructive and destructive interference. If the sample beam and reference beam were exactly parallel to one another then no interference would be detected by the camera’s sensor. If one of the beams was tilted in relation to the other, but the beams intersected at some point in terms of their beam path on the camera’s sensor, then straight interference fringes would be observed. The thickness and tilt of these fringes is influenced by the angle of tilt of one mirror with respect to the other mirror. The interference pattern tilt and thickness can be manipulated by changing the tilt, either up and down or sideways, of one of the mirrors to align with a particular crystal face. As the crystal grows or dissolves the concentration of solution within the resultant boundary layer will vary with distance from the crystal surface into the bulk solution and display different refractive indexes. This causes a bending in the fringes due to the differing path lengths of the beam traveling through the solution near to the crystal surface. The thickness and tilt of the fringes were aligned with the selected face ({120}) of an l-alanine crystal. This can be seen in Figure 5.

Boundary layer thickness was determined using ImageJ by measuring the number of pixels between the crystal surface and the point where the fringes stopped bending, each pixel being 9.6 μm in length.^72^ Boundary layer refractive index distributions were calculated using the equation:
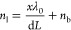
3where *n*_l_ is the refractive index at a chosen point in the boundary layer, *x* (m) is how far the fringe has shifted at a chosen point in the boundary layer compared to the bulk solution fringe (- for dissolution) (Figure 5), λ_0_ (m) is the wavelength of the laser in a vacuum (632.8 nm), *d* (m) is the fringe spacing in the bulk solution (Figure 5), *L* (m) is the length of the crystal surface in the direction parallel to the beam, and *n*_b_ is the refractive index of the bulk solution. Calibrations of refractive index at different concentrations and temperatures allowed determination of the refractive index in the bulk solution. This was performed a number of times within the boundary layer to obtain a plot of refractive index against distance from the crystal surface. Once the refractive index within the boundary layer was determined, extrapolation to a saturation temperature vs refractive index plot, using the relationship between refractive index vs temperature at the saturation temperature of 20 °C, allowed the determination of the saturation temperature at different points in the boundary layer. Comparison to the solubility curve allowed determination of the concentration within the boundary layer.

The viscosity at any point in the boundary layer can be deduced if the concentration and temperature are known. The viscosity at saturation was determined and then extrapolated, using the relationship between viscosity and temperature, to the current temperature of the system for each point in the boundary layer. The diffusion coefficient throughout the boundary layer and bulk solution was then calculated using the Stokes-Einstein equation for diffusion of spherical particles through a liquid with a low Reynolds number:
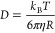
4where *k*_B_ is the Boltzmann constant (1.38064852 × 10^–23^ m^2^ kg s^–2^ K^–1^), *T* is the temperature (K), η is the dynamic viscosity (Pa S), and *R* is the radius of the molecule (2.95 × 10^–10^ m). The radius of the molecule was determined by calculating the volume of the unit cell and dividing it by the number of molecules in the unit cell. The radius could then be calculated by assuming the molecule is spherical. Mass flux (J, kg m^–2^ s^–1^) could then be calculated using Fick’s first law of diffusion:
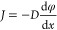
5where d*φ* is the concentration difference (kg m^–3^) and d*x* is the difference in distance (m). Mass flux was calculated at a distance of 30 μm from the crystal surface. A quantitative measure of crystal interfacial kinetics or mass transfer control was determined through calculation of an effectiveness factor for growth and dissolution (η_c_) via the following equation:
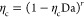
6where Da is the Damkohler number representing the ratio of the mass transfer coefficient to the pseudo-first-order crystal interfacial kinetics coefficient at the bulk conditions and is described by the following equation:
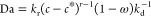
7where ω is mass fraction of solute in solution. *k*_d_ and *k*_r_ are calculated using eq 2. As η_c_ approaches 1 the process is controlled by diffusion, but as it approaches Da^–1^ the process is controlled by the crystal interfacial kinetics.^7,28,29^

## 3 Results and Discussion

### 3.1 Crystal and Solution Properties

Analysis of l-alanine’s morphology revealed a family of {011}, {120}, {110}, and {020} faces (see Figure S3 for the crystal morphology, Table S1 for the interfacial angles, and Supporting Information S7 for more detailed morphology results). The {120} face was selected for further analysis with the interferometer due to its large size and shape allowing the laser to pass over its surface for a significant amount of time and for the same amount of time across much of the face. Modeling of l-alanine’s lattice energy and attachment energies allowed the morphology to be predicted. This morphology showed similarities with experimentally grown crystals (see Figure S4 for the morphology comparison, Table S2 for the attachment energies, and Supporting Information S8 for more detailed modeling results).

The MSZW was found to vary from 17.3 °C at 0.177 g solute mL^–1^ solvent to 20.3 °C at 0.208 g mL^–1^ (see Figure S5). As there was little variation in the MSZW within this range it appeared to be adequate for crystal growth using the concentration and supersaturations studied in this paper (see Supporting Information S9 for more detailed MSZW results). The solubility varied from 14.24 g 100 mL^–1^ at 10 °C to 22.25 g 100 mL^–1^ at 50 °C following a positive exponential trend (see Figure S6 and Supporting Information S10 for more detailed solubility results). The solubility seemed ideal for growing large single crystals and showed reasonable agreement with the literature data and dissolution temperatures measured with the Crystal 16. Viscosity decreased with increasing temperature and decreased with increasing saturation temperature (see Figure S7 and Supporting Information S11).

### 3.2 Refractive Index Calibration

**Figure 6:**
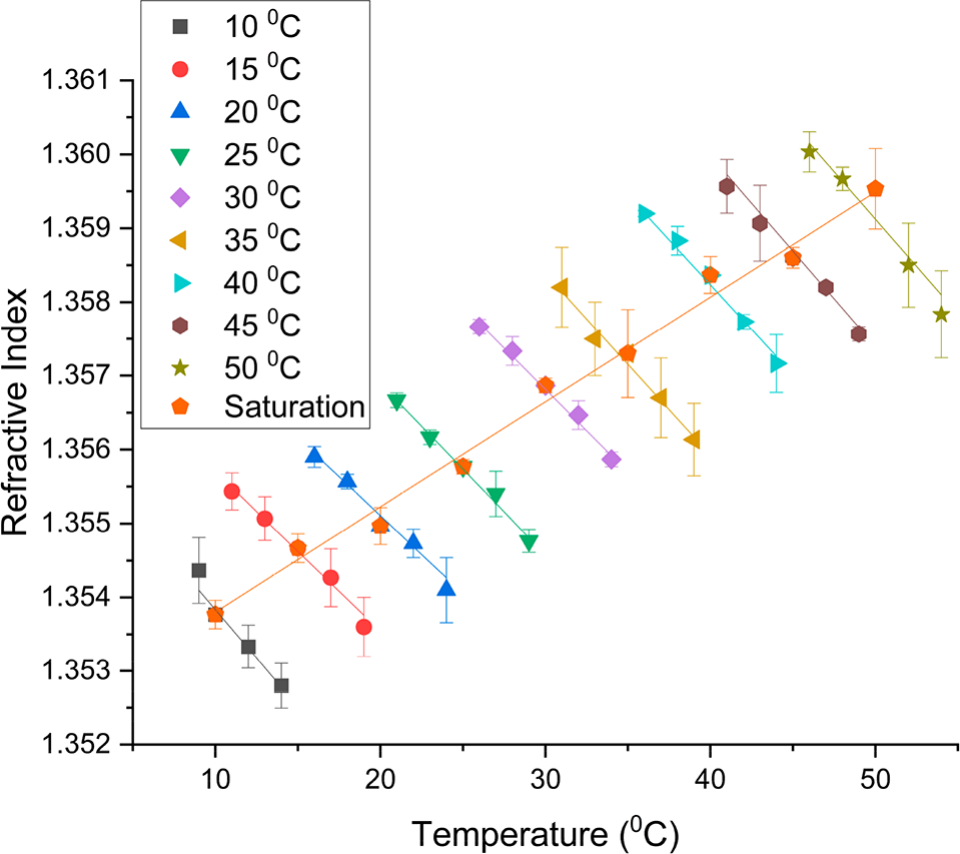
Refractive index of l-alanine in water as a function of saturation temperature and temperature.

Refractive index was measured as a function of saturation temperature and temperature for l-alanine in water. For each saturated solution the dependence on temperature was linear over the temperature range measured and decreased with increasing temperature; this can be seen in Figure 6. There is a positive linear trend as saturation temperature increases. These results were used to calibrate the interferometer and determine the concentration distribution within the boundary layer. Once the refractive index at different points in the boundary layer was determined using eq 3, a plot of refractive index against distance from the crystal surface could be visualized. This can be seen in Figure S8 (see Supporting Information S12 for more details).

**Figure 7:**
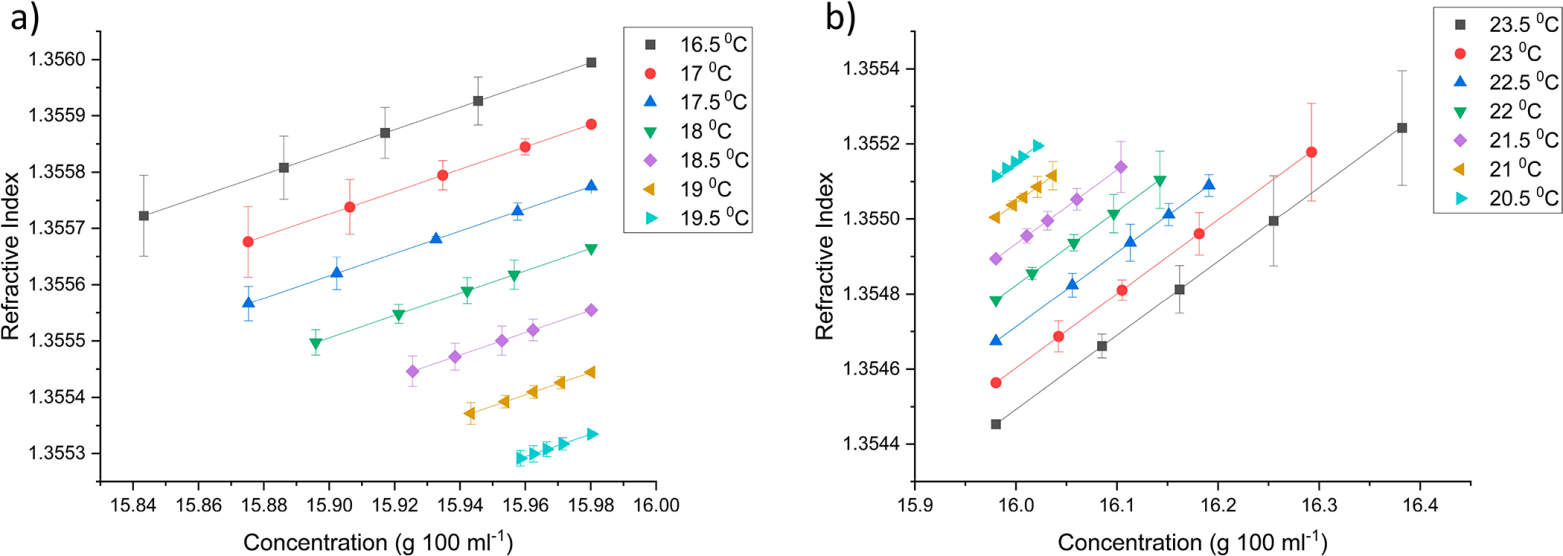
Graphs showing how the calculated refractive index in the boundary layer relates to the solute concentration (g 100 mL^–1^) during growth (a) and dissolution (b) of the {120} face of l-alanine single crystals for different super/undersaturated solutions saturated at 20 °C (0.041/-0.039:16.5/23.5 °C, 0.035/-0.034:17/23 °C, 0.029/-0.028:17.5/22.5 °C, 0.023/-0.023:18/22 °C, 0.017/-0.017:18.5/21.5 °C, 0.012/-0.011:19/21 °C, and 0.006/-0.006:19.5/20 °C).

Once the refractive index within the boundary layer was calculated it was extrapolated to the saturation temperature vs refractive index plot using the relationship between refractive index and temperature at 20 °C (Figure 6). Comparing this saturation temperature to the solubility curve allowed determination of the concentration (Figure S6 and Supporting Information S10). Figure 7 shows this overall calibration process by showing the relationship between the refractive index within the boundary layer and solute concentration at each super/undersaturation. There was a linear relationship at all super/undersaturations between the calculated refractive index and solute concentration. This was due to the same linear dependence on refractive index vs temperature, at the saturation temperature of 20 °C, being used to extrapolate the calculated refractive index to the refractive index vs saturation temperature plot to obtain the temperature that the solute concentration in the boundary layer would be saturated at. The solubility curve shows a relatively small deviation from linearity due to a small concentration change resulting in a linear relationship between the calculated refractive index and solute concentration.

The minimum detectable solute concentration difference was determined revealing the accuracy of this technique in measuring solute concentration in the boundary layer. Generally, the error in the determination of solute concentration was ±0.01 g 100 mL^–1^ although this did increase slightly at higher undersaturations (see Table S3 and Supporting Information S12). Increasing the interference fringe spacing in the bulk solution would increase the accuracy of the Mach–Zehnder interferometer. However, if the fringe spacing is increased too much the uncertainty in determining the fringe position shift also increases.

### 3.3 Boundary Layer Structure

**Figure 8:**
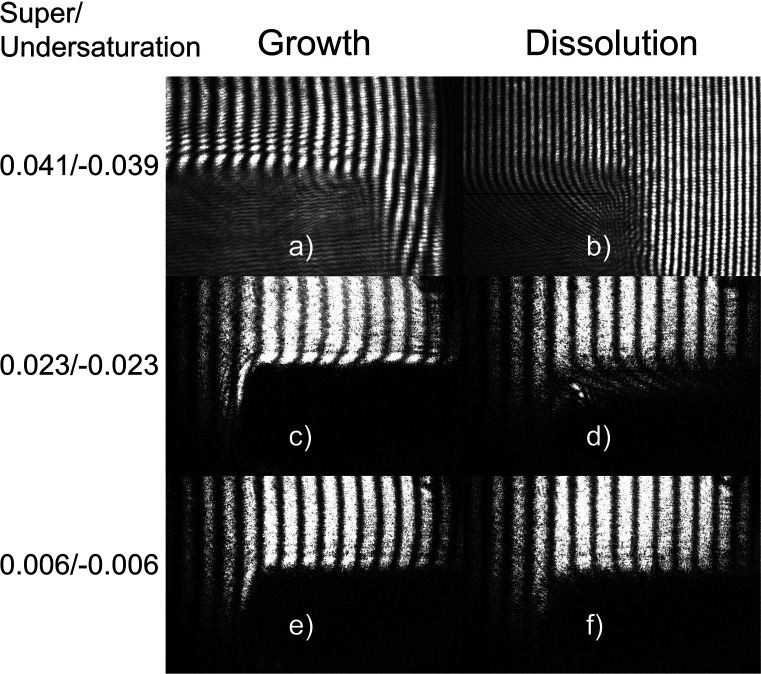
Raw data obtained using the Mach–Zehnder interferometer showing interferograms during growth and dissolution at the super/undersaturations: 0.041/-0.039 (a/b), 0.023/-0.023 (c/d), and 0.006/-0.006 (e/f). The crystal appears as a dark patch in either the bottom left or right of the interferogram. At higher undersaturations the surface is indicated by a horizontal black line.

Figure 8 shows some examples of the interference fringes at 3 super/undersaturations (0.041/-0.039, 0.023/-0.023, and 0.006/-0.006). The bending of the interference fringes became less pronounced as the solution moved toward saturation (see Supporting Information S13 and Figures S9 and S10 for images of the interferograms and details on the interpretation of the images).

#### 3.3.1 {120} Boundary Layer Structure during Growth

**Figure 9:**
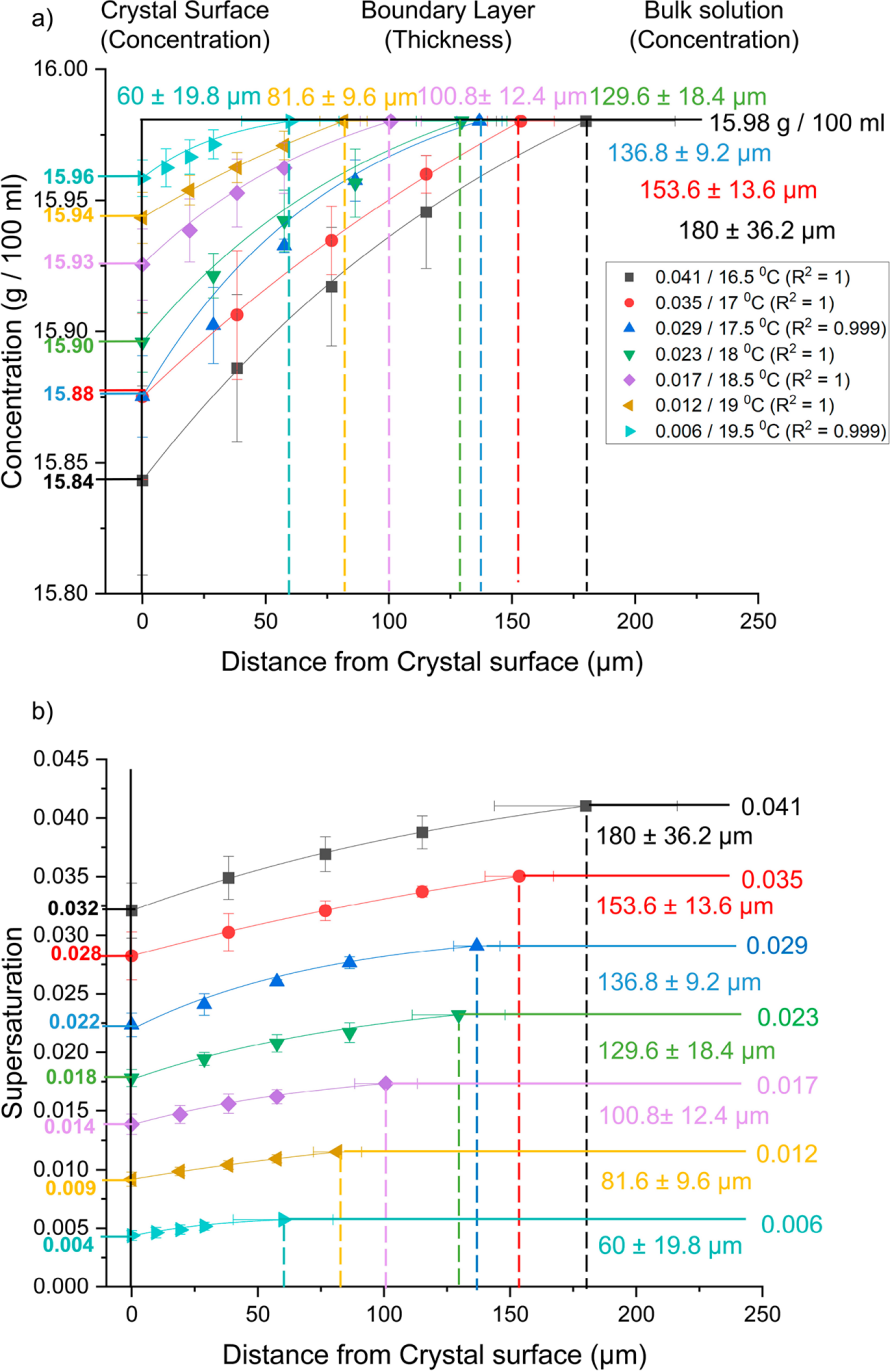
Graphs showing how the concentration (g 100 mL ^–1^) (a) and supersaturation (b) varied with distance from the crystal surface (μm) during growth of the {120} face of l-alanine single crystals for different supersatured solutions saturated at 20 °C (16.5 °C/0.041, 17 °C/0.035, 17.5 °C/0.029, 18 °C/0.023, 18.5 °C/0.017, 19 °C/0.012, and 19.5 °C/0.006) at a flow rate of 0.5 cm^3^ s^–1^. The crystal surface is depicted as a solid black line at 0 μm with surface values for each supersaturation indicated on the left axes of the graphs. Boundary layer thickness is depicted as dashed lines in the middle of the graphs with values indicated in their relative colors. Bulk values are shown in the right region of the graphs as a bold line for (a) and horizontal colored lines for (b).

Figure 9 shows how the concentration (g 100 mL^–1^) (a) and supersaturation (b) varied within the boundary layer during growth of the {120} face of l-alanine single crystals at different supersaturations (0.041, 0.035, 0.029, 0.023, 0.017, 0.012, and 0.006) and characterizes the boundary layer thickness, solute concentration distribution, and the supersaturations at the crystal interface, as a function of the solution bulk supersaturation. Table 1 also shows what the surface and bulk values are as well as the boundary layer thickness.

**Table 1:** Table Showing How the Bulk/Surface Concentration, Supersaturation, and Boundary Layer Thickness Changed with Temperature for the {120} Face of l-Alanine during Growth

Temperature (°C)	16.5	17	17.5	18	18.5	19	19.5
Bulk Concentration (g 100 mL^–1^)	15.98	15.98	15.98	15.98	15.98	15.98	15.98
Surface Concentration (g 100 mL^–1^)	15.84 *± 0.04*	15.88 *± 0.03*	15.88 *± 0.02*	15.90 *± 0.01*	15.93 *± 0.01*	15.94 *± 0.01*	15.96 *± 0.01*
Bulk Supersaturation	0.041	0.035	0.029	0.023	0.017	0.012	0.006
Surface Supersaturation	0.032 *± 0.002*	0.028 *± 0.002*	0.022 *± 0.001*	0.018 *± 0.001*	0.014 *± 0.001*	0.009 *± 0.001*	0.004 *± 0.001*
BL Thickness (μm)	180 *± 36.2*	153.6 *± 13.6*	136.8 *± 9.2*	129.6 *± 18.4*	100.8 *± 12.4*	81.6 *± 9.6*	60 *± 19.8*

It is true for all supersaturations on the {120} face that the concentration within the boundary layer decreased from the bulk concentration toward the crystal surface (Figure 9a). This is due to the integration of solute molecules onto the {120} crystal surface and the resultant concentration gradient which drives the mass transfer of solute molecules from solution to the crystal-solution interface. At the bulk supersaturation of 0.041 it can be seen that bulk concentration was 15.98 g 100 mL^–1^, but the surface concentration was 15.84 g 100 mL^–1^, so the concentration at the surface had decreased (Figure 9a and Table 1).

**Figure 10:**
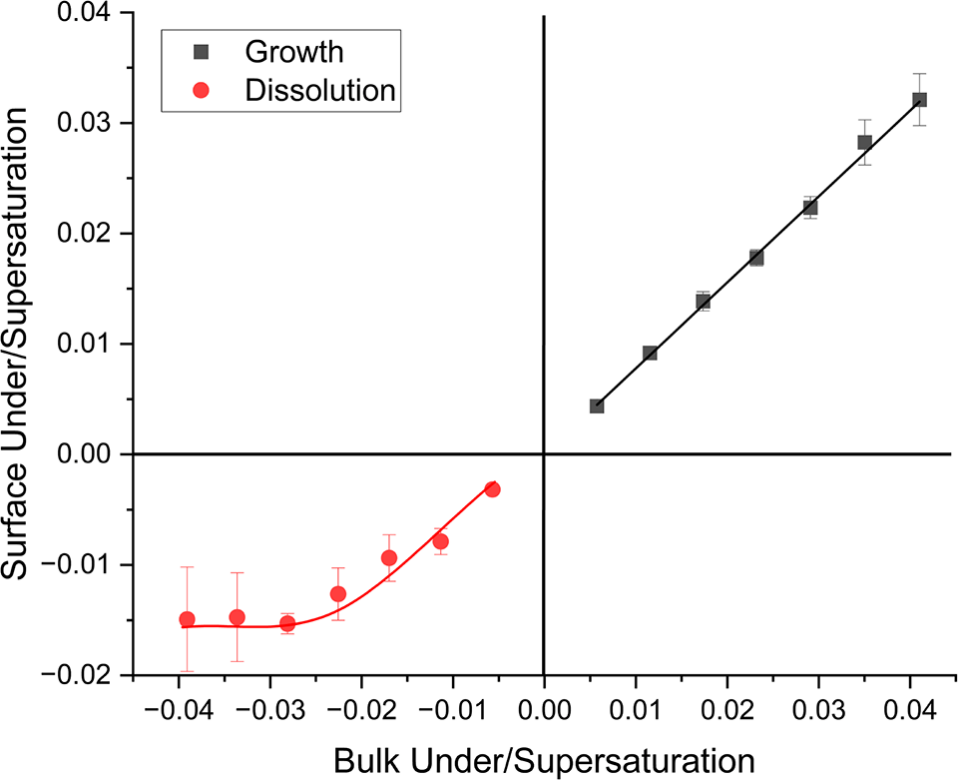
Graph showing how surface under/supersaturation changed with bulk under/supersaturation during dissolution and growth of the {120} face of l-alanine.

In Figure 10, as the bulk supersaturation increased the supersaturation at the crystal surface increased linearly, and, hence, the growth rate would increase. The concentration at the crystal surface was always higher than the equilibrium concentration at the temperature that the solution was supersaturated (Figure 9a, b and Table 1). In the extreme case, if the concentration at the crystal surface was the same as the equilibrium concentration the surface supersaturation would be 0. This means that growth is faster than the rate of mass transfer so the mass transfer through the boundary layer would be the limiting step and control the overall rate of growth. On the other hand, if the surface supersaturation was closer to the supersaturation of the bulk, then the opposite would be true and crystal growth would be limited more by the crystal interfacial kinetics. This indicates that the crystal interfacial kinetics limits the growth rate on these faces to some degree.^8,24^ There was an increased difference between the surface and the bulk supersaturation as the bulk supersaturation increased, which was due to an increased concentration difference between the crystal surface and bulk solution. As the bulk supersaturation increases the solution becomes more unstable increasing the driving force for crystal growth. The relative ratio between the surface and bulk supersaturation did not change showing a linear relationship as the bulk supersaturation increased.^24^

**Figure 11:**
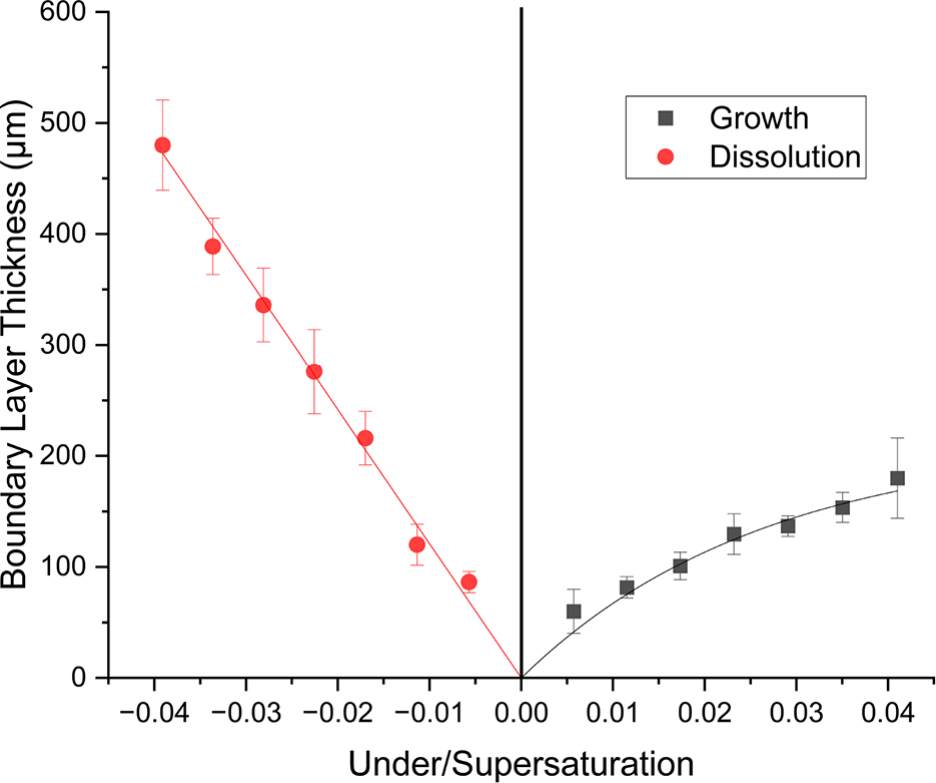
Relationship between boundary layer thickness and bulk under/supersaturation during dissolution growth of the {120} face of an l-alanine single crystal.

According to the boundary layer observation of barium nitrate crystal growth in the literature, the boundary layer thickness increased as supersaturation increased in a range between 200 and 400 μm.^22^ The boundary layer was also observed in the literature during growth at a flow rate of 3 cm s^–1^ where it was observed that the thickness was ∼50–75 μm at a supersaturation of 4.88%.^24^ The flow rate of 3 cm s^–1^ used in the literature was higher than the flow rate used in this research, which was 0.04 cm s^–1^. The boundary layer observed here during growth increased nonlinearly with increasing bulk supersaturation (Figures 9 and 11 and Table 1). This is caused by an increased mass flux of solute molecules through the boundary layer as the bulk supersaturation increased. An equilibrium is created between the mass flux through the boundary layer and integration of solute molecules into the crystal lattice determining the surface concentration and subsequent dependence of crystal growth on either mass transfer or crystal interfacial kinetics. The boundary layer thickness is clearly associated with not only the concentration difference between the crystal surface and bulk solution, but also the mass flux of solute molecules through the boundary layer, which in turn is affected by the viscosity and the subsequent diffusion coefficient, as well as the supersaturation.

#### 3.3.2 {120} Boundary Layer Structure during Dissolution

**Figure 12:**
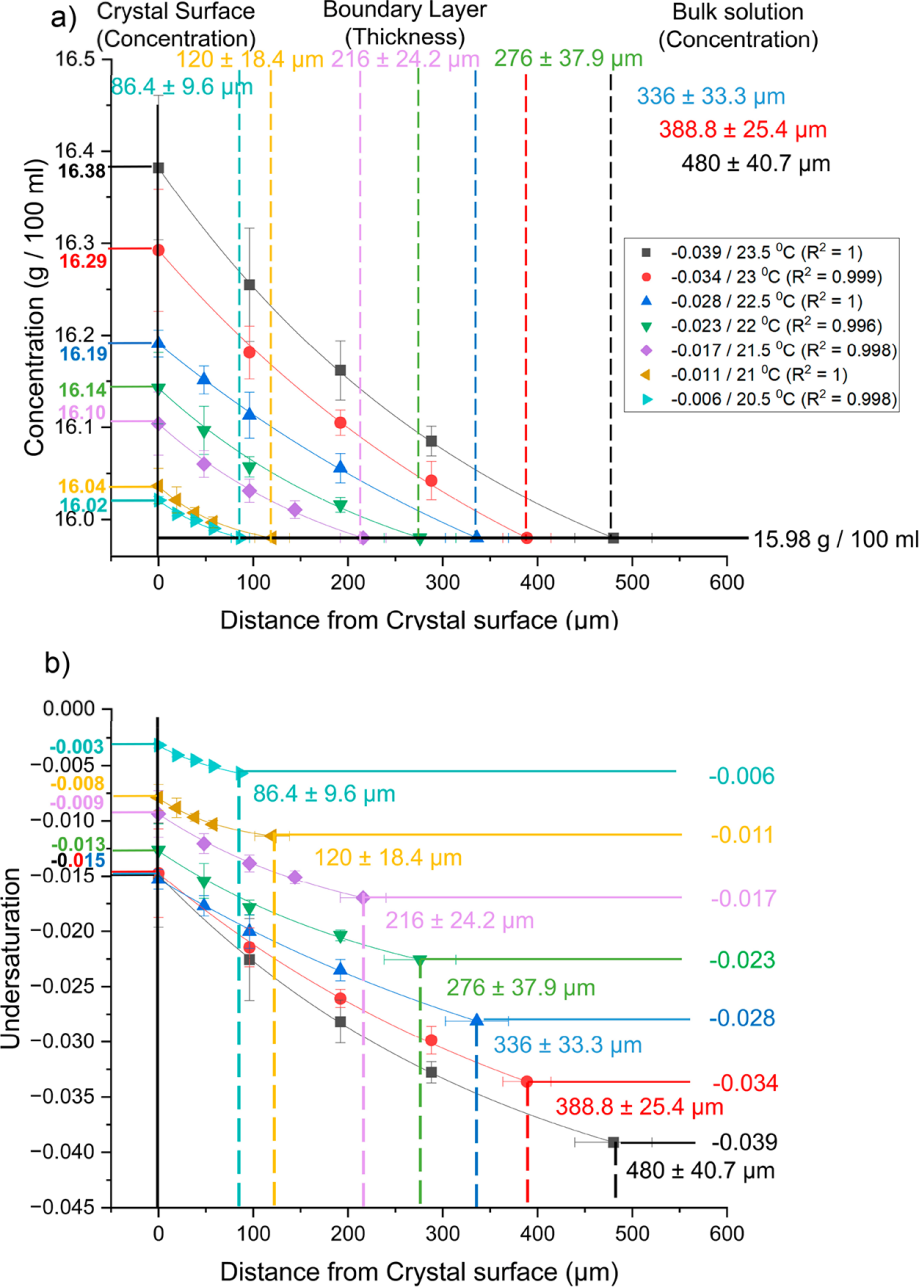
Graphs showing how the concentration (g 100 mL^–1^) (a) and undersaturation (b) varied with distance from the crystal surface (μm) during dissolution of the {120} face of l-alanine single crystals for different undersaturated solutions saturated at 20 °C (23.5 °C/-0.039, 23 °C/-0.034, 22.5 °C/-0.028, 22 °C/-0.023, 21.5 °C/-0.017, 21 °C/-0.011, and 20.5 °C/-0.006) at a flow rate of 0.5 cm^3^ s^–1^. The crystal surface is depicted as a solid black line at 0 μm with surface values for each undersaturation indicated on the left axes of the graphs. Boundary layer thickness is depicted as dashed lines in the middle of the graphs with values indicated in their relative colors. Bulk values are shown in the right region of the graphs as a bold line for (a) and horizontal colored lines for (b).

Figure 12 shows how the concentration (g 100 mL^–1^) (a) and undersaturation (b) varied with distance from the crystal surface (μm) during dissolution of the {120} face of l-alanine single crystals at different undersaturations (−0.039, −0.034, −0.028, −0.023, −0.017, −0.011, and −0.006). It characterizes the boundary layer thickness and solution concentration and undersaturation distribution within the boundary layer as a function of the solution bulk undersaturation. Table 2 summarizes what the surface and bulk values are as well as the boundary layer thickness.

**Table 2:** Table Showing How the Bulk/Surface Concentration, Undersaturation, and Boundary Layer Thickness Changed with Temperature for the {120} Face of l-Alanine during Dissolution

Temperature (°C)	23.5	23	22.5	22	21.5	21	20.5
Bulk Concentration (g 100 mL^–1^)	15.98	15.98	15.98	15.98	15.98	15.98	15.98
Surface Concentration (g 100 mL^–1^)	16.38 *± 0.08*	16.29 *± 0.07*	16.19 *± 0.01*	16.14 *± 0.04*	16.10 *± 0.03*	16.04 *± 0.02*	16.02 *± 0.01*
Bulk Undersaturation	–0.039	–0.034	–0.028	–0.023	–0.017	–0.011	–0.006
Surface Undersaturation	–0.015 *± 0.005*	–0.015 *± 0.004*	–0.015 *± 0.001*	–0.013 *± 0.002*	–0.009 *± 0.002*	–0.008 *± 0.001*	–0.003 *± 0.002*
BL Thickness (μm)	480 *± 40.7*	388.8 *± 25.4*	336 *± 33.3*	276 *± 37.9*	216 *± 24.2*	120 *± 18.4*	86.4 *± 9.6*

During dissolution the concentration decreased from the crystal surface to the bulk solution as solute molecules diffused into the bulk solution. This can be seen for all undersaturations studied on the {120} face (Figure 12a). This is the opposite effect to what is seen during growth and is due to the detachment of molecules from the crystal lattice caused by the undersaturated state of the solution increasing the concentration of molecules at the surface of the crystal. The solute molecules then diffused into the bulk solution through the boundary layer. This resulted in a higher concentration at the surface of the crystal compared to the bulk solution. If the concentration was lower this would indicate slower detachment of solute molecules from the crystal lattice and *vice versa*. Looking at the −0.039 bulk undersaturation it can be seen that the bulk concentration was 15.98 g 100 mL^–1^, but the surface concentration was 16.38 g 100 mL^–1^, so the concentration at the surface was higher (Figure 12a and Table 2).

Similarly to growth, as the bulk undersaturation increases the driving force for dissolution increases, and so the dissolution rate would also increase, increasing the detachment of solute molecules from the surface of the crystal and increasing the surface concentration. The concentration at the crystal surface, however, was always lower than the equilibrium concentration at the temperature that the solution was undersaturated at for all of the bulk undersaturations studied. This indicates that the crystal interfacial kinetics were slow enough to limit the dissolution rate somewhat on these faces which is somewhat contradictory to diffusion based models used to describe dissolution such as the Noyes-Whitney equation.^9,10^

It can be seen that at the same relative super/undersaturation levels the concentration difference between the surface and the bulk was always larger for dissolution than for growth. (Figure 9a/12a and Table 1/2). At the super/undersaturation of 0.023 the concentration difference was 0.16 g 100 mL^–1^ for the {120} face during dissolution but 0.08 g 100 mL^–1^ during growth. This alone indicates that detachment of solute molecules from the surface is faster than attachment to the surface under the same relevant conditions of super/undersaturation. This can be explained by factors that make dissolution a faster process than growth (i.e., the increased number of active sites from edge dislocations and point defects during dissolution, impurities in the crystal lattice slowing growth but speeding up dissolution, the increased activities of etch pits compared to growth hillocks, etc.).^20−24^ It could also be a function of the surface chemistry and the differences in interaction between the surface and solution during growth and dissolution. It is also clear that this concentration difference became more accentuated at higher super/undersaturations. It is said beyond a certain undersaturation the formation of Type I etch pits increases the dissolution rate.^24^

It is clear from this data that dissolution and growth are not symmetrical processes, and dissolution is faster than growth in this instance for the {120} face.^7,19^ The impact of crystal interfacial kinetics on dissolution indicates that diffusional processes do not solely limit dissolution. This is in contrast with what is stated in the Noyes-Whitney equation and is concurrent with recent data on dissolution rates being different for different morphological faces of APIs.^9,10,12,13^ As bulk supersaturation increased during growth surface supersaturation increased in a linear manner. During dissolution surface undersaturation appeared to increase linearly initially and then remained at the same value for undersaturations larger than −0.023 (Figure 10). Below this undersaturation surface concentration must start to increase at a faster rate leading to a lower relative undersaturation at the surface compared to the bulk. It is also clear that the surface was much closer to saturation during dissolution than growth in all instances. This gives some indication that crystal interfacial kinetics is slower in growth than dissolution, although it must have some effect on the dissolution rate.

The boundary layer observed here increased with increasing bulk undersaturation and was dependent on the same factors as during growth (Figure 11). The boundary layer was much larger during dissolution than during growth to a factor of 1.4 at the lowest bulk super/undersaturations to 2.7 at the largest bulk super/undersaturations for the {120} face. This difference can be associated with differences in surface concentration, surface to bulk concentration differences, and increased diffusion coefficient during dissolution due to the increased temperature. During dissolution, the concentration at the surface was higher creating a larger concentration difference between the surface and the bulk solution and subsequently a thicker boundary layer. Boundary layer thickness also appeared to increase linearly with increasing undersaturation during dissolution. This may be attributed to the rapidly increasing surface concentration during dissolution as undersaturation increased which may have proportionally increased the boundary layer thickness so that its trend was linear with bulk undersaturation.

### 3.4 Diffusion Coefficient

**Figure 13:**
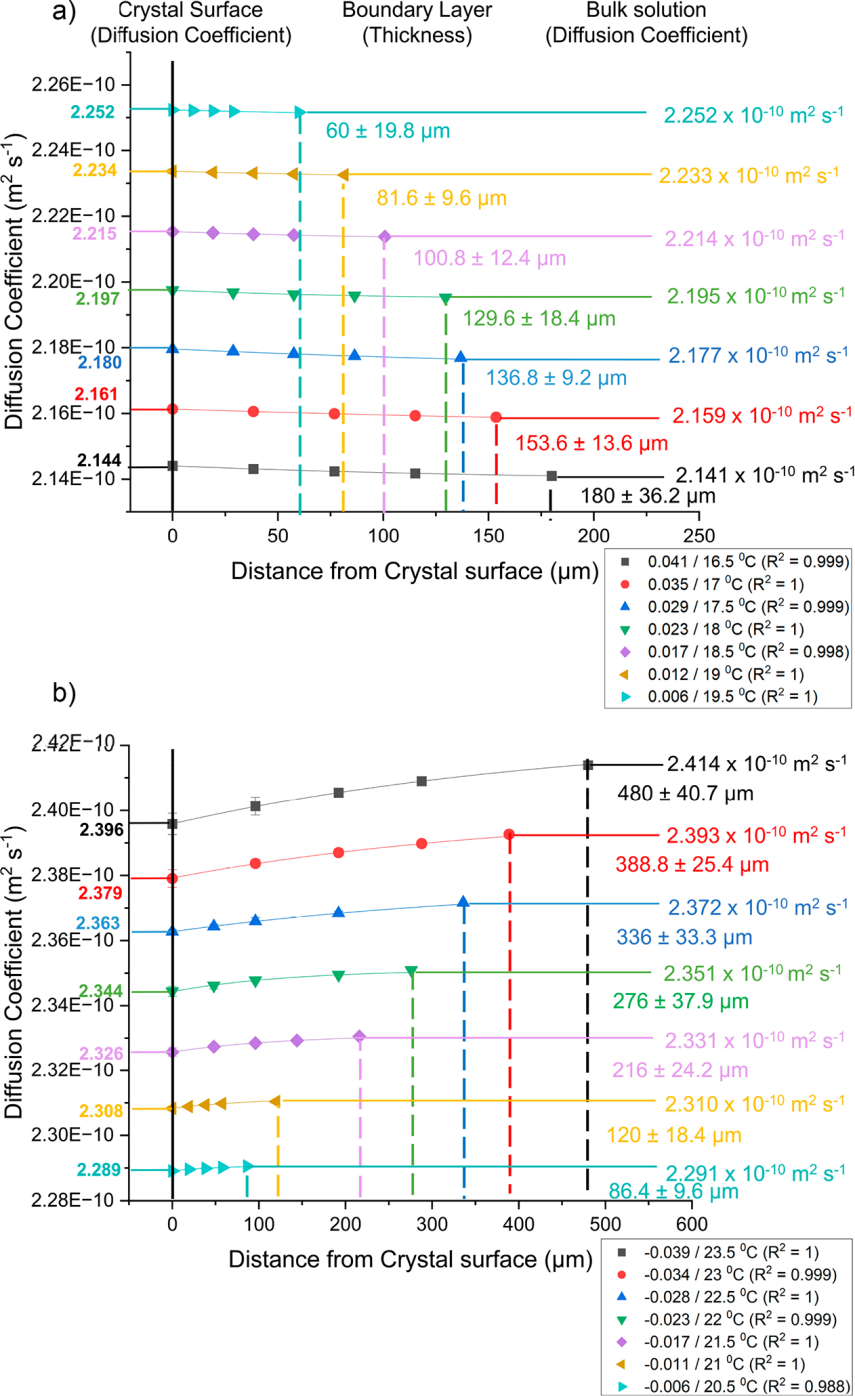
Graphs showing how the diffusion coefficient (m^2^ s^–1^) varied with distance from the crystal surface (μm) during growth (a) and dissolution (b) of the {120} face of l-alanine single crystals at different super/undersaturations (0.041/-0.039, 0.035/-0.034, 0.029/-0.028, 0.023/-0.023, 0.017/-0.017, 0.012/-0.011, and 0.006/-0.006) at a flow rate of 0.5 cm^3^ s^–1^. The crystal surface is depicted as a solid black line at 0 μm with surface values for each super/undersaturation indicated on the left axes of the graphs. Boundary layer thickness is depicted as dashed lines in the middle of the graphs with values indicated in their relative colors. Bulk values are shown in the right region of the graphs as horizontal colored lines.

Figure 13 shows how the diffusion coefficient (m^2^ s^–1^) varied with distance from the crystal surface (μm) during growth (a) and dissolution (b) of the {120} face of l-alanine single crystals at different super/undersaturations (0.041/-0.039, 0.035/-0.034, 0.029/-0.028, 0.023/-0.023, 0.017/-0.017, 0.012/-0.011, and 0.006/-0.006). The diffusion coefficient was calculated using the Stokes–Einstein equation (eq 4). It characterizes the boundary layer thickness and diffusion coefficient distribution within the boundary layer as a function of the solution bulk super/undersaturation. Table 3 also shows what the surface and bulk values are.

**Table 3:** Table Showing How the Bulk/Surface Diffusion Coefficient Changed with Temperature and Bulk Super/Undersaturation for the {120} Face of l-Alanine during Growth and Dissolution

Temperature (°C)	16.5/23.5	17/23	17.5/22.5	18/22	18.5/21.5	19/21	19.5/20.5
Bulk Super/Undersaturation	0.041/-0.039	0.035/-0.034	0.029/-0.028	0.023/-0.023	0.017/-0.017	0.012/-0.011	0.006/-0.006
Bulk Diffusion Coefficient Growth (m^2^ s^–1^) 10^10^	2.141	2.159	2.177	2.195	2.214	2.233	2.252
Surface Diffusion Coefficient Growth (m^2^ s^–1^) 10^10^	2.144 *± 0.001*	2.161 *± 0.001*	2.180 *± 0.001*	2.197 *± 0.001*	2.215 *± 0.001*	2.234 *± 0.001*	2.252 *± 0.001*
Bulk Diffusion Coefficient Dissolution (m^2^ s^–1^) 10^10^	2.414	2.393	2.372	2.351	2.331	2.310	2.291
Surface Diffusion Coefficient Dissolution (m^2^ s^–1^) 10^10^	2.396 *± 0.003*	2.379 *± 0.003*	2.363 *± 0.001*	2.344 *± 0.002*	2.326 *± 0.001*	2.308 *± 0.001*	2.289 *± 0.001*

The diffusion coefficient decreased slightly from the surface of the crystal to the bulk solution during growth (Figure 13a). This was due to an increase in concentration which increased viscosity and subsequently decreased the diffusion coefficient. The increased difference between the surface and bulk diffusion coefficient as bulk supersaturation increased was due to the larger concentration difference between the surface of the crystal and the bulk solution. As the temperature increased supersaturation decreased and the diffusion coefficient increased. This was not only caused by the viscosity decreasing as temperature increased, but it was also the result of the diffusion coefficient being directly dependent on the temperature. It seems to be the case that the diffusion coefficient was influenced more by the temperature of the system rather than the concentration of the system in the experiments observed here.

The diffusion coefficient increased from the surface of the crystal to the bulk solution during dissolution (Figure 13b). Conversely to growth, this was due to a decrease in concentration which decreased viscosity and subsequently increased the diffusion coefficient. The increased difference between the surface and bulk diffusion coefficient as bulk undersaturation increased was due to the larger concentration difference between the surface of the crystal and the bulk solution. As the temperature increased undersaturation increased and the diffusion coefficient increased. When comparing this to growth similar dependencies are seen. The difference being that as temperature increased during dissolution the undersaturation increased but during growth the supersaturation decreased.

### 3.5 Mass Flux

**Figure 14:**
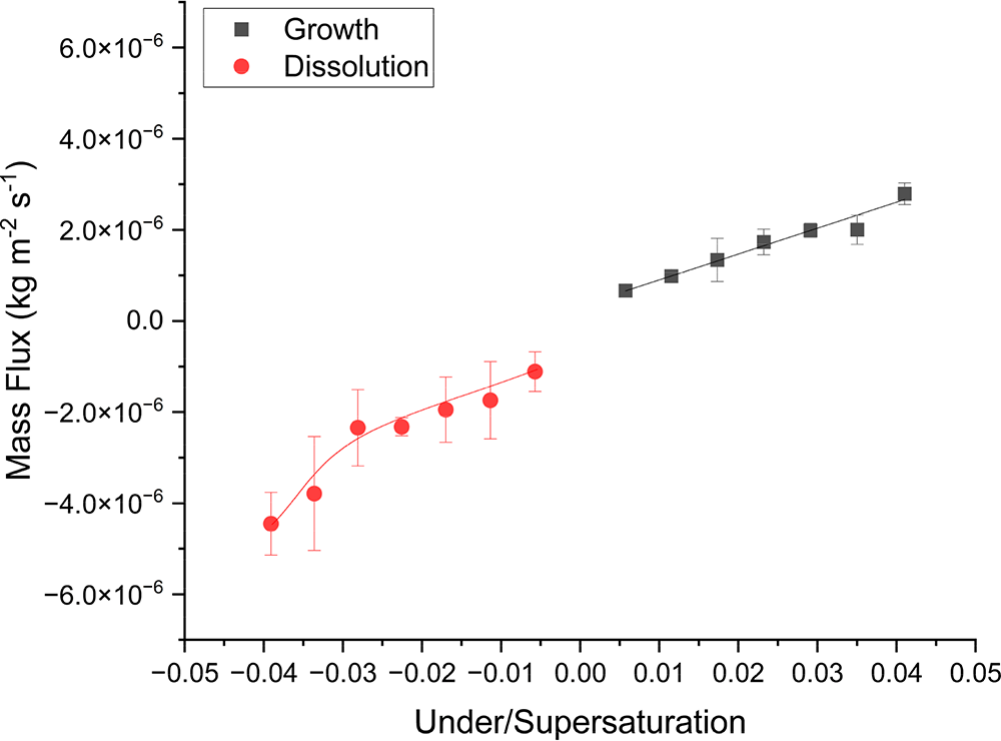
Graph showing how mass flux changed with undersaturation and supersaturation during dissolution and growth of the {120} face of l-alanine.

Figure 14 shows how the mass flux (kg m^–2^ s^–1^) varied during growth and dissolution of the {120} face of l-alanine single crystals at different super/undersaturations (0.041/-0.039, 0.035/-0.034, 0.029/-0.028, 0.023/-0.023, 0.017/-0.017, 0.012/-0.011, and 0.006/-0.006) at a flow rate of 0.5 cm^3^ s^–1^. The mass flux was calculated using Fick’s first law of diffusion (eq 5) from the surface of the crystal up to a distance of 30 μm from the crystal surface. As supersaturation and undersaturation increased, the mass flux increased (Figure 14 and Table 4). This was primarily caused by the increasing difference in concentration between the surface of the crystal and the bulk solution relative to the boundary layer thickness. As in, the concentration difference increased faster than the boundary layer thickness increased as super/undersaturation increased. It appears, at these particular under/supersaturations observed that the increase in mass flux with super/undersaturation was almost linear in nature apart from at undersaturations larger than −0.028. The boundary layer thickness did not increase as fast as the concentration difference due to the increased under/supersaturation in the bulk solution creating a more unstable solution increasing the driving force for crystal growth/dissolution and, indeed, the mass flux. The sharp increase in the mass flux at undersaturations larger than −0.028 is concurrent with the sharp increase in the concentration difference between the crystal surface and bulk solution beyond this undersaturation.

**Table 4:** Values of the Mass Flux and Effectiveness Factor at Different Super/Undersaturations during Growth and Dissolution for the {120} Face of l-Alanine

Temperature (°C)	16.5/23.5	17/23	17.5/22.5	18/22	18.5/21.5	19/21	19.5/20.5
Bulk super/undersaturation	0.041/-0.039	0.035/-0.034	0.029/-0.028	0.023/-0.023	0.017/-0.017	0.012/-0.011	0.006/-0.006
Mass flux growth (kg m^–2^ s^–1^) 10^6^	2.793 *± 0.238*	2.002 *± 0.321*	1.989 *± 0.153*	1.733 *± 0.280*	1.340 *± 0.475*	0.984 *± 0.042*	0.666 *± 0.069*
Mass flux dissolution (kg m^–2^ s^–1^) 10^6^	–4.452 *± 0.690*	–3789 *± 1.249*	–2.346 *± 0.839*	–2.323 *± 0.200*	–1.948 *± 0.717*	–1.742 *± 0.847*	–1.113 *± 0.433*
Effectiveness factor for growth	0.81	0.83	0.79	0.79	0.82	0.82	0.79
Effectiveness factor for dissolution	0.42	0.47	0.58	0.60	0.59	0.72	0.59

The mass flux during dissolution was generally larger than the mass flux during growth for the {120} face at the same relative under/supersaturation. This can be attributed to the higher concentration difference between the surface and bulk during dissolution. So, this must indicate that the boundary layer thickness is associated with the concentration difference as well as the under/supersaturation. Another interesting phenomenon was that the diffusion coefficient increased as the temperature increased. During growth, as the supersaturation increased, the temperature was decreasing, and so, the diffusion coefficient decreased. During dissolution, as the undersaturation increased, the temperature was increasing and the diffusion coefficient increased. So, as the supersaturation increased during growth, although the concentration gradient was increasing, the diffusion coefficient was decreasing, so the difference in the mass flux became less pronounced. During dissolution, as the undersaturation increased, the concentration gradient and diffusion coefficient were increasing. This, in theory, would increase the mass flux to a greater degree and decrease the boundary layer thickness relative to the concentration difference having a more pronounced effect on the mass flux during dissolution. In any case, it can be seen in Figure 14 that the trend in the mass flux with undersaturation was steeper during dissolution than growth. This shows that dissolution rates would be faster than growth rates in these instances at the same relative under/supersaturation.

Through calculation of an effectiveness factor (using eqs 6 and 7), it was determined whether growth and dissolution were controlled by either the crystal interfacial kinetics or mass transfer within the boundary layer.^7,28,29^ It was found that at all supersaturations that growth was limited by crystal interfacial kinetics to the same degree with the effectiveness factor varying from 0.79 to 0.83 (Table 4). This means that as the supersaturation increased the relative impact of crystal interfacial kinetics and mass transfer on the growth rate remained the same. Dissolution was found to have a mixed dependency on crystal interfacial kinetics and mass transfer (Table 4). The effectiveness factor initially remained at the same value, with the exception of the −0.011 undersaturation, and varied within the range of 0.58–0.60. At undersaturations larger than −0.028 the dissolution process started to become more limited by mass transfer through the boundary layer. This was also observed in the rapid increase in concentration above this undersaturation and the leveling off of the surface undersaturation as bulk undersaturation increased as well as the sharp increase in the mass flux (Table 2/4 and Figure 10/14). It is clear that dissolution can be controlled by crystal interfacial kinetics to some degree, and this was particularly true at lower undersaturations.

## 4 Conclusion

The crystal–aqueous solution interface for the {120} face of l-alanine has been characterized using Mach–Zehnder interferometry in situ during growth and dissolution experiments resulting in an assessment of the boundary layer concentration distribution, thickness, diffusion coefficient, and mass flux. The measurement of this study revealed that increasing the super/undersaturation increased the concentration difference between the surface and the bulk solution. As a result of this, the boundary layer thickness increased, but this increase was offset by the increased mass flux of solute molecules within the boundary layer. During growth and dissolution, the solution concentration at the surface was found to not be saturated indicating that crystal interfacial kinetics must have been slow enough to impact the growth and dissolution rates for the {120} face. This is contrary to current diffusion based models for dissolution. The concentration differences during dissolution were far larger than during growth. This was also reflected in the boundary layer thickness and surface undersaturation.

The diffusion coefficient was found to decrease as concentration increased due to an increase in solution viscosity but as temperature increased it increased rapidly. The mass flux increased with increasing super/undersaturation. This was associated with the increased concentration difference between the crystal surface and bulk solution. It was also faster for dissolution than for growth at the same relative under/supersaturation. Growth was limited by crystal interfacial kinetics at all supersaturations to the same degree whereas dissolution displayed a mixed dependency on mass transfer and crystal interfacial kinetics at lower undersaturations but became more limited by mass transfer at higher undersaturations.

This study shows that in certain circumstances dissolution can be limited by both crystal interfacial kinetics and mass transfer. Due to the obscure nature of the crystal interfacial kinetic coefficient, and many growth and dissolution models being based on bulk solution properties, more work needs to be done to elucidate the link between the surface integration/detachment of solute molecules into/from the crystal lattice and the surface chemistries of crystal faces. Mass flux calculations and boundary layer structure determination using this Mach–Zehnder interferometry methodology provides the first step in overcoming current limitations of growth and dissolution models. Combining the mass flux calculations and boundary layer structure on different crystal surfaces with accurate growth and dissolution kinetics measured with a Michelson interferometer will provide a deeper insight into how the surface chemistry affects growth and dissolution. Further work needs to be done to elucidate how the boundary layer structure differs on different crystal surfaces and how this is related to their surface chemistries through molecular modeling. An improved model for growth and dissolution will have a huge impact in the chemical industry, as well as closely related areas, in terms of improving the fundamental understanding of both dissolution and growth as well as providing a basis for accurately predicting growth and dissolution rates without the need for extensive experimentation. It may also allow poorly soluble APIs to be designed to overcome current issues with bioavailability. This overall project aims to develop new face-specific growth and dissolution models based upon the above criteria.

## Supporting Information Available

A method for growing large single crystals is presented in S1. The method for the analysis of the morphology from the large single crystals of l-alanine is shown in S2. The method for the modeling of l-alanine’s lattice energy and attachment energies using Habit98 as well as visualizing the resultant morphology using Mercury, to corroborate the experimentally determined morphology results, is presented in S3. The method for determining the MSZW using polythermal crystallization is presented in S4. Beyond this, the method for the calibration of the interferometer using the solubility (S5) and viscosity (S6) are presented. The results and discussion are also presented for l-alanine’s experimental morphology, modeled morphology, MSZW, solubility, viscosity and refractive index (sections S7, S8, S9, S10, S11, and S12, respectively). Finally, raw data in the form of interferograms is displayed for 4 runs of l-alanine single crystals growing and dissolving in water at various super/undersaturations in S13. (PDF)

## Dedication

Dedicated to the life and works of Professor John N. Sherwood.

## Abbreviations

2-D: Two-Dimensional
3-D: Three-Dimensional
AFM: Atomic Force Microscopy
API: Active Pharmaceutical Ingredient
BCS: Biopharmaceutical Classification System
BFDH: Bravais-Friedel-Donnay-Harker
BL: Boundary Layer
CSD: Cambridge Structural Database
GA: Gravimetric Analysis
GI: Gastrointestinal
He–Ne: Helium–Neon
LR: Linear Regression
MSZW: Metastable Zone Width
RI: Refractive Index
SICM: Scanning Ion-Conductance Microscopy.

## Symbols

*A*: surface area of crystal face, m
*c*: solution concentration, kg m^–3^
*c*_i_: concentration at crystal surface, kg m^–3^
*c*^***^: equilibrium solution concentration, kg m^–3^
*D*: diffusion coefficient, m^2^ s^–1^
Da: Damkohler number
*d*: interference fringe spacing in the bulk solution, m
*d*_*hkl*_: interplanar spacing, Å
d*m*: change in mass, kg
d*t*: change in time, s
d*x*: change in distance, m
d*φ*: change in concentration, kg m^–3^
*g*: order of the overall growth process
*J*: mass flux, kg m^–2^ s^–1^
*K*_G_: overall growth coefficient, m s^–1^
*k*_B_: Boltzmann constant, 1.380649 × 10^–23^ m^2^ kg s^–2^ K^–1^
*k*_d_: mass transfer coefficient, m s^–1^
*k*_r_: crystal interfacial kinetic coefficient, m s^–1^
*L*: length of crystal surface in contact with laser beam, m
*n*_b_: refractive index in the bulk solution
*n*_l_: refractive index in the boundary layer
*R*: molecular radius, m
*R*_G_: growth rate, kg m^–2^ s^–1^
*r*: order of crystal interfacial kinetics
*T*: temperature, K
*T*_cryst_: temperature of crystallization, K
*T*_diss_: temperature of dissolution, K
*x*: the distance the interference fringe has shifted compared to the interference fringe in the bulk solution, m
δ: boundary layer thickness, m
η: dynamic viscosity, Pa S
η_c_: effectiveness factor
λ_0_: wavelength of the laser beam in a vacuum, m
σ: relative super/undersaturation
ω: mass fraction of solute in solution
