# Supplementary material for: Characterization of Mass Transfer within the Crystal-Solution Boundary Layer of l-Alanine {120} Faces Using Laser Interferometry during Growth and Dissolution
Source: Cryst Growth Des. 2023 Mar 16;23(4):2755–69. doi: 10.1021/acs.cgd.2c01541 (PMC10080658; doi:10.1021/acs.cgd.2c01541)
Supplement: Supplementary file 1 — cg2c01541_si_001.pdf [file cg2c01541_si_001.pdf]

Supporting Information: Characterization of Mass Transfer within the Crystal-Solution Boundary Layer of L-alanine {120} Faces using Laser Interferometry during Growth and Dissolution

*Steven T. Nicholson[1], Kevin J. Roberts[1], Toshiko Izumi[2] and Xiaojun Lai[1]**

1 EPSRC Centre for Doctoral Training in Complex Particulate Products and Processes, School of Chemical and Process Engineering, University of Leeds, Woodhouse Lane, Leeds, LS2 9JT, U.K

2 Pfizer R&D UK, Ramsgate Road, Sandwich, Kent, CT13 9NJ, U.K

* Corresponding Author: X.Lai@leeds.ac.uk

Dedicated to the life and works of Professor John N. Sherwood

Key Words:

Crystallization, Growth, Dissolution, Boundary Layer, Interferometry, Mach-Zehnder, Diffusion Coefficient, Mass Flux, Mass Transfer, L-alanine, Single Crystal

ABSTRACT

This document contains supporting information related to the research article - Characterization of Mass Transfer within the Crystal-Solution Boundary Layer of L-alanine {120} Faces using Laser Interferometry during Growth and Dissolution.

A method for growing large single crystals is presented in S1. The method for the analysis of the morphology from the large single crystals of L-alanine is shown in S2. The method for the modeling of L-alanine's lattice energy and attachment energies using Habit98 as well as visualizing the resultant morphology using Mercury, to corroborate the experimentally determined morphology results, is presented in S3. The method for determining the MSZW using polythermal crystallization is presented in S4. Beyond this, the method for the calibration of the interferometer using the solubility (S5) and viscosity (S6) are presented. The results and discussion are also presented for L-alanine's experimental morphology, modeled morphology, MSZW, solubility, viscosity and refractive index (sections S7, S8, S9, S10, S11 and S12 respectively). Finally, raw data in the form of interferograms is displayed for 4 runs of L-alanine single crystals growing and dissolving in water at various super/undersaturations in S13.

S1. Large Single Crystal Growth Method

Once adequate seed crystals were grown, they was prepared for further crystal growth to determine their morphology. To ensure there was even growth on all faces, the crystal was suspended in a supersaturated solution. A 0.3 mm hole was 'drilled' through the crystal, using subtle twisting movement and water, and a 0.2 mm piece of fishing line fed through the hole to attach it to a glass impeller.

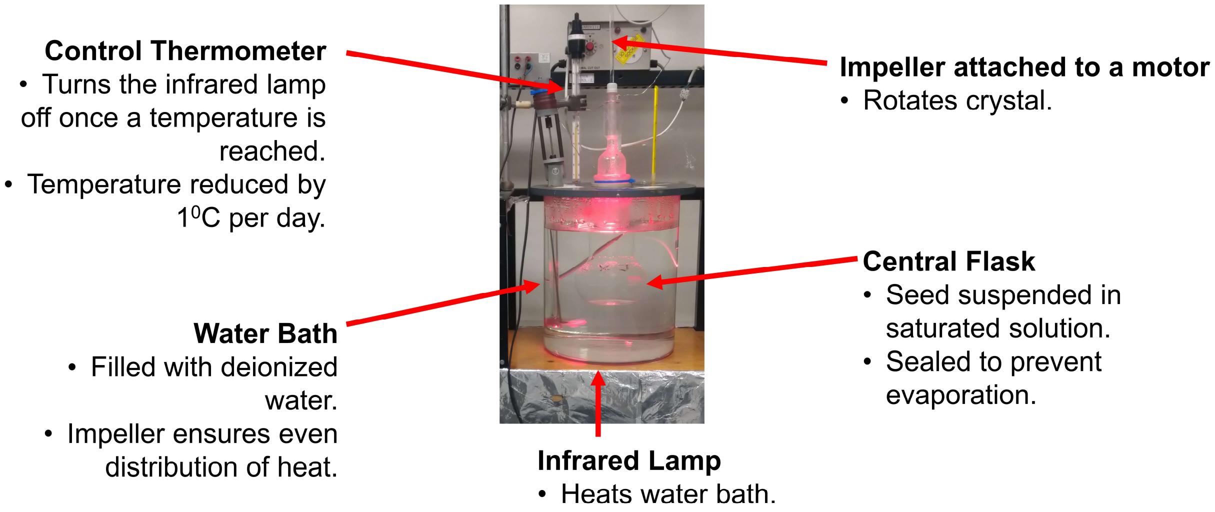

Figure S1. Setup of the rig used for growth of large single crystals.

In order to grow crystals of large sizes efficiently, specialized crystal growth apparatus was employed and can be seen in Figure S1.[1] A flask with a greaseless vacuum seal and mercury seal stirrer was used. An external water bath was heated using an infrared lamp controlled by a thermistor. There was sufficient agitation to ensure even distribution of heat across the whole crystal growth vessel. The glass impeller stirred the internal solution with the seed crystal attached at 30 rpm.

A saturated solution was prepared at 40 ^{0}C. This solution was transferred into the central flask and placed into the water bath which was heated to 42 ^{0}C. The seed crystal was suspended within the saturated solution at 42 ^{0}C before reducing the temperature to 39 ^{0}C once the crystal had been

introduced into the solution. This initial stage where the solution was undersaturated ensured that the rough outer layers of the seed crystal were dissolved. The temperature was lowered by 0.5 ^{0}C in the morning and then in the evening each day for 3 weeks yielding crystals with dimensions ~3 x 1 x 1 cm^3.

S2. Morphology Method

The experimental morphology was measured using a single crystal ~3 x 1 x 1 cm^3 in size grown from solution using the large single crystal growth rig. A ruler and protractor were used to measure the interfacial angles between the crystal faces. These angles were combined with unit cell parameters to index each of the faces with their lowest possible miller indices.[2]

S3. Crystal Chemistry Modeling

The LALNIN12 structure was downloaded from the CSD in Mercury.[2,3] The partial atomic charges were calculated using Mopac.[4] The Momany force field potential set was chosen for calculation of the lattice energy. This potential set uses a 6-12 potential but also an additional 10-12 general hydrogen bonding potential.[5] The lattice energy was calculated at various distances from 3-30 Å using Habit98.[6]

The morphology of L-alanine was predicted by calculation of the attachment energies of potential faces. The attachment energies were calculated using the FULL mode of Habit98 by listing a series of potential faces selected using the Bravais-Friedel-Donnay-Harker (BFDH) rule, literature data and experimentally observed morphology.[6-11] The attachment energy is the partition of the lattice energy used to attach a slice of thickness d_{hkl} onto another slice in a particular morphological direction. The faces determined in the experimental morphology were generally those that had the lowest attachment energies and these were plotted in Mercury to give the predicted morphology.[3]

S4. Polythermal Crystallization

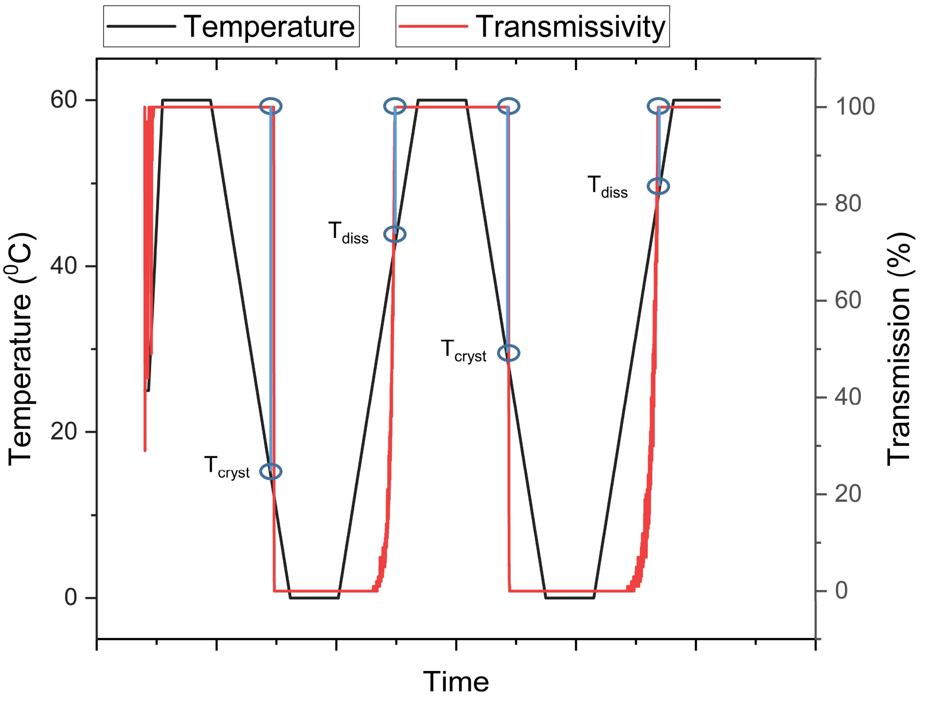

Figure S2. An example of the polythermal crystallization method indicating how the temperature and transmittance changes with time as well as how T_{diss} and T_{cryst} were calculated.

Polythermal crystallization was carried out using a Technobis Crystal 16 unit. This unit contains 16 reactors where 4 different temperature profiles can be run at once for 4 samples each. Solutions are heated and cooled using Peltier heated aluminum blocks, a water bath and a nitrogen line. Solutions of L-alanine in water were prepared at 0.177, 0.188, 0.198 and 0.208 g ml^{-1}. Each concentration was run at 4 heating/cooling rates: 0.1, 0.2, 0.3 and 0.5 ^{0}C min^{-1} from 1 ^{0}C to 60 ^{0}C and *vice versa* with the solutions being stirred at 700 rpm. The temperature profiles ramped up and down 3 times for each sample. This revealed the average temperatures of crystallization and dissolution through measurement of the transmission through the solution. The temperature profile and transmissivity profile can be seen in Figure S2.

When the transmission reached 100 % this indicated all of the crystalline material had dissolved (T_{diss}). When it dropped below 100 % this showed crystallisation had started (T_{cryst}). Average values of T_{diss} and T_{cryst} for each concentration were plotted against cooling/heating rates. Extrapolation to a 0 ^{0}C min^{-1} cooling/heating rate revealed the equilibrium T_{diss} and T_{cryst} values. The MSZW was calculated from the difference between T_{diss} and T_{cryst}.

S5. Solubility Method

Solubility was measured using gravimetric analysis (GA). This involved preparing saturated solutions of L-alanine in water at 5 ^{0}C intervals between 10 ^{0}C and 50 ^{0}C. Vials were weighed, shaken for 24 hours in an incubated shaker with an excess of solute in solvent, filtered to remove excess solute, weighed with the saturated solution, left in an oven at 50 ^{0}C for ~ a week and then weighed with just the solute until the weight remained constant.

The relative super/undersaturation (σ) of the system was calculated using the following equation:

$$\sigma = \frac{c - c^*}{c^*} \qquad \text{S1}$$

Where c is the solution concentration ($kg\ m^{-3}$) and c^* is the equilibrium solution concentration ($kg\ m^{-3}$).

S6. Viscosity Method

Viscosity was measured using an Anton Paar Physica MCR301 Rheometer. Solutions were prepared in the same way as the gravimetric analysis method except they were prepared at 5 ^{0}C intervals between 10 ^{0}C and 35 ^{0}C. Saturated solutions were pipetted into the cup and bob geometry of the Rheometer. The temperature was kept undersaturated in the Rheometer at first, 4 ^{0}C above the saturation temperature, and viscosity measurements were taken every 1 ^{0}C, down to 4 ^{0}C below the saturation temperature. At each temperature 5 measurements were taken 10 seconds apart. The

solution was allowed to equilibrate at each temperature for 3 minutes before each measurement was taken. These values were taken at a shear rate of 400 s^{-1}.

S7. Morphology

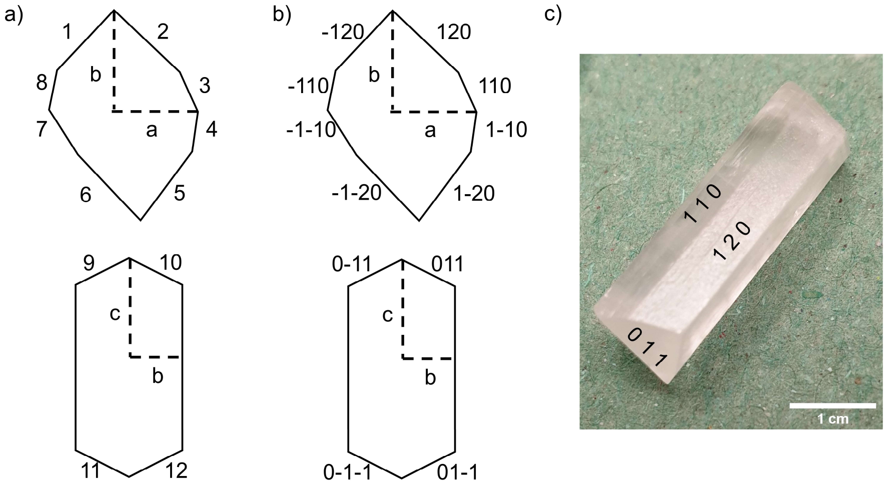

Figure S3. Schematic of the crystal looking down the c-axis and a-axis without indexing (a), with indexing (b) and an actual indexed L-alanine single crystal (c).

Each observed face of L-alanine was initially given a number (Figure S3a). The angles between each of the faces were measured. These measurements are in Table S1. These angles were then combined with unit cell parameters (a = 6.032 Å, b = 12.343 Å, c = 5.784 Å) to index each of the faces.[2] This was done to minimize the miller indices of each of the faces revealing a family of {120}, {110} and {011} faces (Figure S3b/c). Two small {020} faces were also observed between the {120} family of faces (Figure S3c). This is concurrent with morphologies shown in the literature.[7-10]

Table S1. Interfacial angles measured between the faces of an L-alanine single crystal.

**Indices**	**Angle Measured (^{0})**
1:2	90
2:3	155
3:4	125
4:5	155
5:6	90
6:7	155
7:8	125
8:1	155
Edge:9/10/11/12	112
9:10, 11:12	125

S8. Modeling of L-alanine's Morphology

L-alanine's calculated lattice energy was -33.62 kcal mol^{-1}. This corresponded well with experimentally calculated lattice energies and previously calculated lattice energies in literature.[10]

The attachment energies of the selected faces present in the experimental morphology can be found in Table S2. The lower the attachment energy, the slower the growth and the more significant the face. The morphology was plotted and compared against the experimental morphology in Figure S4.

Table S2. The attachment of potential faces for potential faces of L-alanine.

Miller Indices	Attachment Energy (kcal mol^{-1})
**{120}**	-10.98
**{020}**	-9.10
**{110}**	-9.58
**{011}**	-16.16

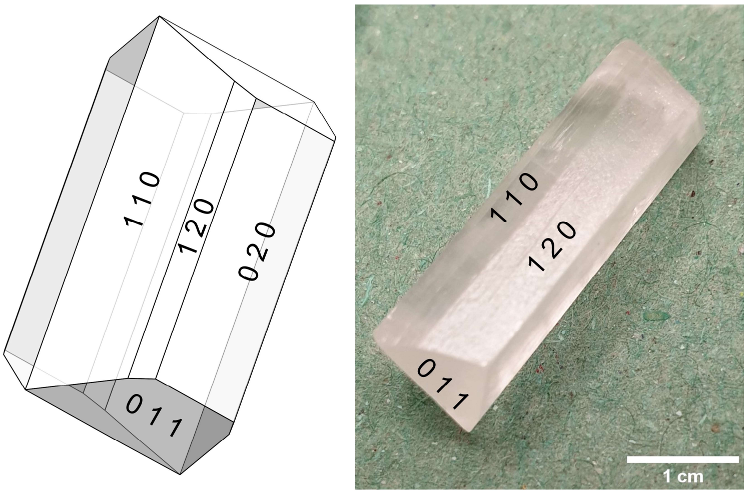

Figure S4. Comparison of the predicted morphology (left) and the experimental morphology (right) for L-alanine.

It can be seen in Figure S4 that there is good agreement between predicted and experimentally grown crystal morphology. The differences arise from differences in growth brought about by the supersaturation level of the solution or interaction of the faces with the solution.

S9. MSZW

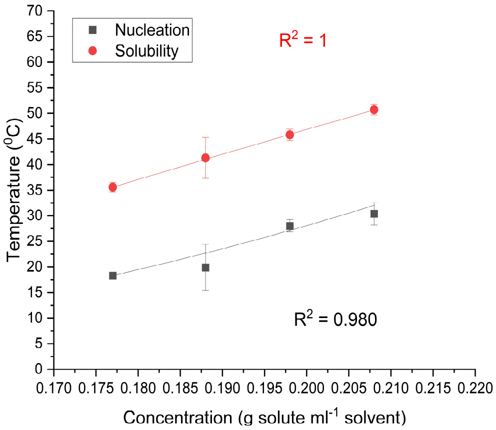

Figure S5. MSZW determination obtained from extrapolation to a 0 ^{0}C min^{-1} cooling/heating rate for 4 concentrations (0.177, 0.188, 0.198 and 0.208 g solute ml^{-1} solvent) showing the solubility curve (T_{diss}), nucleation curve (T_{cryst}) and the resultant MSZW.

The MSZW was determined at 4 concentrations and can be seen in Figure S5. It was found that the MSZW varied from 17.3 ^{0}C at 0.177 g solute ml^{-1} solvent to 20.3 ^{0}C at 0.208 g ml^{-1}. This shows that as concentration increased the MSZW increased slightly. Similar values have been reported previously.[12,13] The MSZW at 20 ^{0}C would be expected to be similar to this due to the low variation over the temperature studied here. So, the MSZW appears to be adequate for single crystal growth using the supersaturations studied in this paper and experiments are kept well within the MSZW.

S10. Solubility

The solubility data for L-alanine in water obtained *via* gravimetric analysis follows a positive exponential trend from 14.24 g solute 100 ml^{-1} solvent at 10^{0}C to 22.25 g 100 ml^{-1} at 50 ^{0}C. This corresponds well with solubility measured previously in literature and can be seen in Figure S6.[14-19] Although the solubility increases with temperature, it does not increase at too fast a rate. This indicates that any small variation in temperature will not drastically affect the growth of the

crystals. Its solubility and temperature dependence are large enough to grow large crystals for use with the interferometer *via* the slow evaporation mechanism and for growing single crystals using the large single crystal growth rig.[1] Solubility data from the Crystal 16 using linear regression (LR) also showed reasonable agreement with the gravimetric analysis data and literature data. Subsequently, the GA data was used for the Mach-Zehnder interferometer analysis.

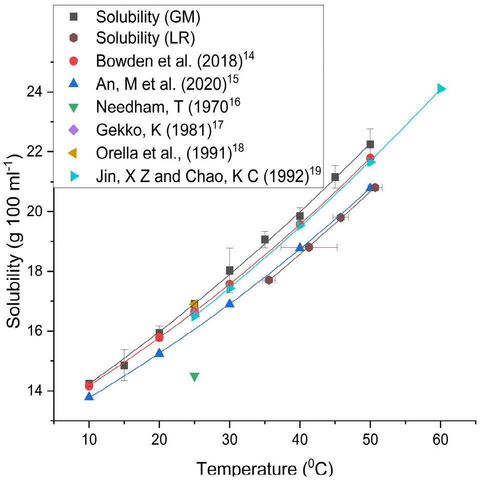

Figure S6. Experimentally measured solubility of L-alanine in water using gravimetric analysis (GA) and linear regression (LR) with comparison to literature data.[14-19]

S11. Viscosity

Viscosity showed an exponential decrease as the saturation temperature increased, this can be seen in Figure S7. Varying the temperature of the solution displayed a linear dependence of viscosity on temperature at each concentration. The decrease in viscosity as saturation temperature increases is most likely due to an increase in temperature of the system as viscosity is more dependent on the temperature here than the concentration. It was found at the same temperature that an increase in concentration would increase the viscosity.

From this data it was possible to deduce what the viscosity was at each point in the boundary layer from the Mach-Zehnder interferogram. If the concentration in the boundary layer is known it is possible to deduce what the viscosity is at the temperature the experiment is currently at by extrapolation from the saturation temperature curve using the dependence of viscosity on temperature.

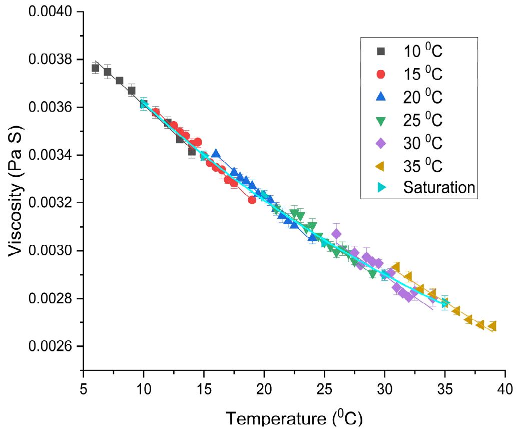

Figure S7. The viscosity of L-alanine in water as a function of saturation temperature and temperature.

S12. Refractive Index

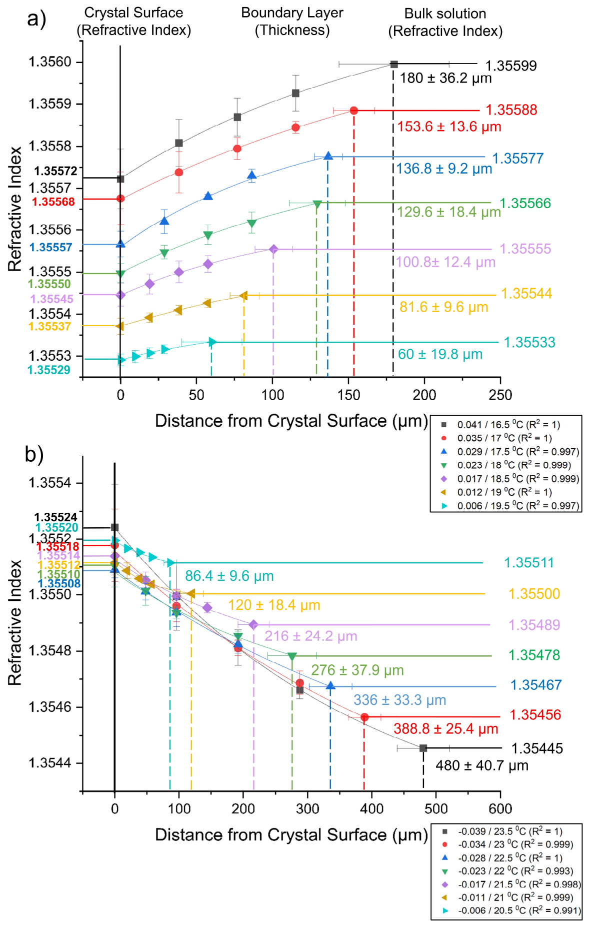

Figure S8. Graphs showing how the refractive index varied with distance from the crystal surface (µm) during growth (a) and dissolution (b) of the {120} face of L-alanine single crystals for different super/undersaturated solutions saturated at 20 ^{0}C (0.041/-0.039, 0.035/-0.034, 0.029/-0.028, 0.023/-0.023, 0.017/-0.017, 0.012/-0.011 and 0.006/-0.006). The crystal surface is depicted as a solid black line at 0 µm with surface values for each super/undersaturation indicated on the left axes of the graphs. Boundary layer thickness is depicted as dashed lines in the middle of the graphs with values indicated in their relative colors. Bulk values are shown in the right region of the graphs as horizontal colored lines.

Figure S8 shows how the refractive index varied within the boundary layer during growth and dissolution of the {120} face of L-alanine single crystals at different super/undersaturations (0.041/-0.039, 0.035/-0.034, 0.029/-0.028, 0.023/-0.023, 0.017/-0.017, 0.012/-0.011 and 0.006/-0.006).

As it is the case that the boundary layer structure is discussed in detail in the main text (section 3.3) a brief explanation about the refractive index distribution will be discussed here. Refractive indexes in the bulk solution are the relative refractive index values at the different temperatures of the 20 ^{0}C temperature vs refractive index plot in Figure 6. Refractive index decreases towards the crystal face during growth due to a decrease in solute concentration and the opposite effect is seen during dissolution. As the temperature of the system increases the refractive index decreases. In the case of dissolution, the decrease in refractive index because of the temperature increase is offset by the increased concentration in the boundary layer as undersaturation increases causing the line fits to cross one another.

It was the case that at the most extreme super/undersaturations the fringe spacing in the bulk solution was smaller than at higher super/undersaturations resulting in a larger error. The largest error was ± 0.08 g 100 ml^{-1} at the -0.039 undersaturation. However, the smallest error was ± 0.006 g 100 ml^{-1} at the -0.006 supersaturation, but these errors could also be due to variation of the boundary layer structure between the four interferometer runs which were averaged to get this error. Table S3 shows the average minimum detectable solute concentration difference at each super/undersaturation associated with the spatial resolution of the camera. This variance can mainly be attributed to differences in the spacing of the interference fringes in the bulk solution. The minimum detectable solute concentration difference was around the same at each super/undersaturation apart from at higher undersaturations. The bending of the fringes was more

easily observable at higher undersaturations so the spacing of the interference fringes in the bulk solution was, in general, lower. Overall, increased fringe spacing in the bulk solution would help with the fringe position reading. Ensuring the fringe spacing in the bulk solution was efficiently maximized would result in a smaller minimum detectable solute concentration difference. However, this increased fringe spacing increases the uncertainty in the fringe position read. The other factor influencing the minimum detectable solution concentration change is the reproducibility associated with the laser beam and optical system mechanical stability (*i.e.* vibration). By repeating the measurements a number of time (*i.e.* 4 times), the mechanical variation effect is reduced.

Table S3. Minimum detectable solute concentration difference (g 100 ml^{-1}) at each super/undersaturation.

**Super/ Undersaturation**	**0.041/ -0.039**	**0.035/ -0.034**	**0.029/ -0.028**	**0.023/ -0.023**	**0.017/ -0.017**	**0.012/ -0.011**	**0.006/ -0.006**
Minimum detectable concentration difference (g 100 ml^{-1}) Growth	± 0.007	± 0.006	± 0.008	± 0.006	± 0.008	± 0.007	± 0.006
Minimum detectable concentration difference (g 100 ml^{-1}) Dissolution	± 0.024	± 0.020	± 0.012	± 0.009	± 0.011	± 0.008	± 0.007

S13. Interferograms

Interferograms were taken of the interference fringes aligned with the {120} face of L-alanine and also of the sample beam without fringes. These can be seen in Figures S9 for growth and S10 for dissolution. It was appropriate in some instances to combine both images to get a better understanding of where the crystal surface may be. During growth, determination of the crystal

surface was more obvious as the fringes became obscured by the presence of the crystal. However, during dissolution, above 20.5 ^{0}C, the location of the crystal surface becomes less obvious. This could stem from the increasing concentration at the crystal surface as undersaturation increased decreasing the contrast between the solution in the boundary layer and the crystal: the refractive index of the crystal and solution within the boundary layer became similar during dissolution at higher undersaturations. The surface in these instances was determined by locating the point where the intensity of light in the interferogram started increasing into the bulk solution and is indicated by a black horizontal line in the dissolution interferograms excluding some of the 20.5 ^{0}C images.

Each super/undersaturation (0.041/-0.039, 0.035/-0.034, 0.029/-0.028, 0.023/-0.023, 0.017/-0.017, 0.012/-0.011 and 0.006/-0.006) was observed 4 times with 4 different crystals. Initially, runs covered the super/undersaturations from 0.029/-0.028 to 0.006/-0.006 so additional runs were performed at the larger super/undersaturations with different crystals. Sometimes it was also apt to redo some of the super/undersaturations. This is why the images within the runs look different in some instances. The {120} face length varied between the crystals slightly. For run 1 the 2 {120} lengths were 1.2 and 1.3 mm. For run 2: 1.3 and 1.1 mm. For run 3: 0.7 and 0.9 mm. For run 4: 1.1 and 1.1 mm. During dissolution it became obvious that the crystal surface was retreating slightly as the undersaturation increased due to slight dissolution of the crystal surface. Thus, the length of the {120} face must have changed as well. This was calibrated and taken into account during the analysis of the interferograms.

S13.1. Growth Interferograms

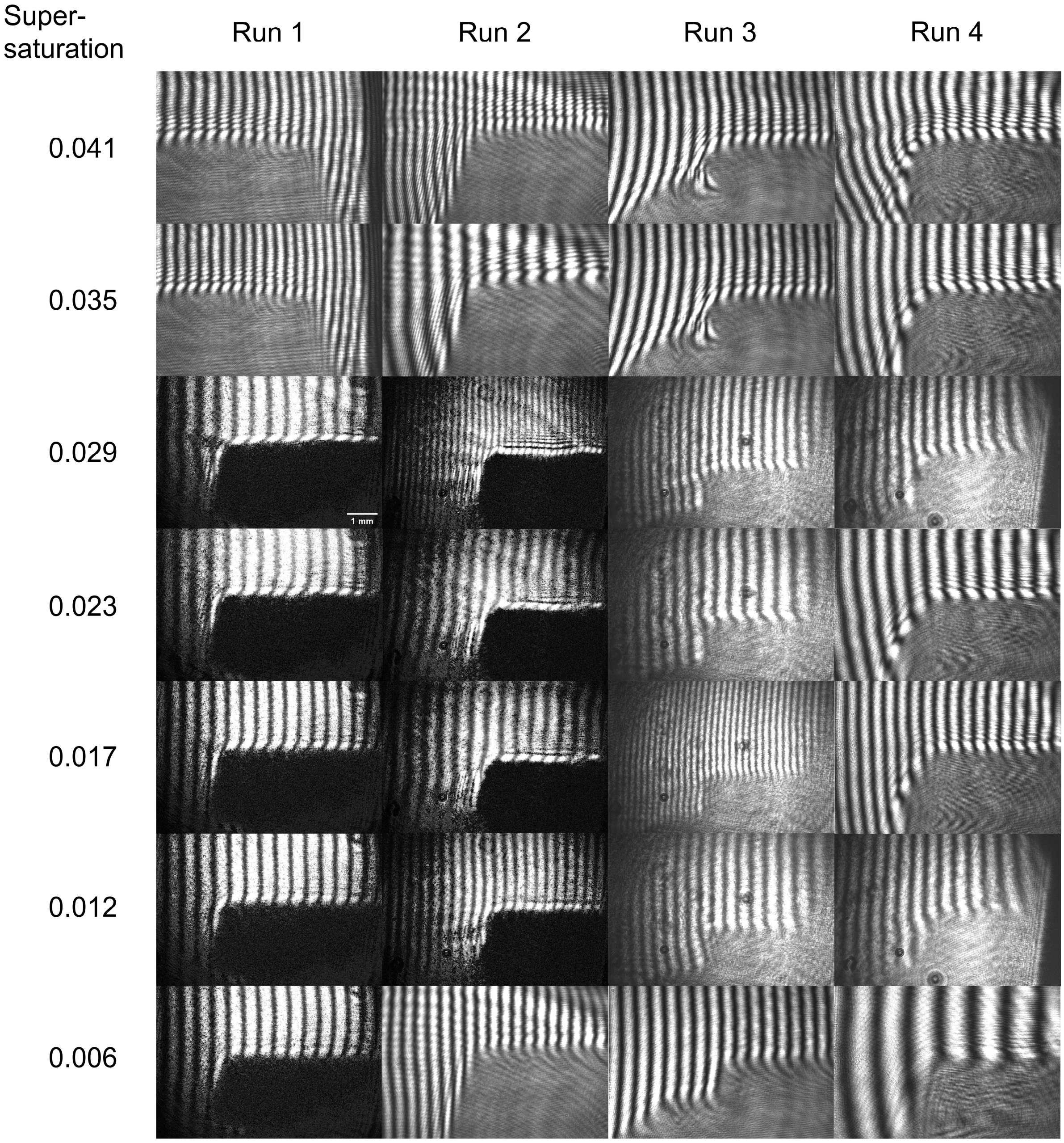

Figure S9. The 4 runs of interferograms of L-alanine growing in water at 16.5 ^{0}C/0.041, 17 ^{0}C/0.035, 17.5 ^{0}C/0.029, 18 ^{0}C/0.023, 18.5 ^{0}C/0.017, 19 ^{0}C/0.012 and 19.5 ^{0}C/0.006. Fringes are aligned perpendicular to the {120} crystal surface and all images have the same scale as in Run 1 0.029.

S13.2. Dissolution Interferograms

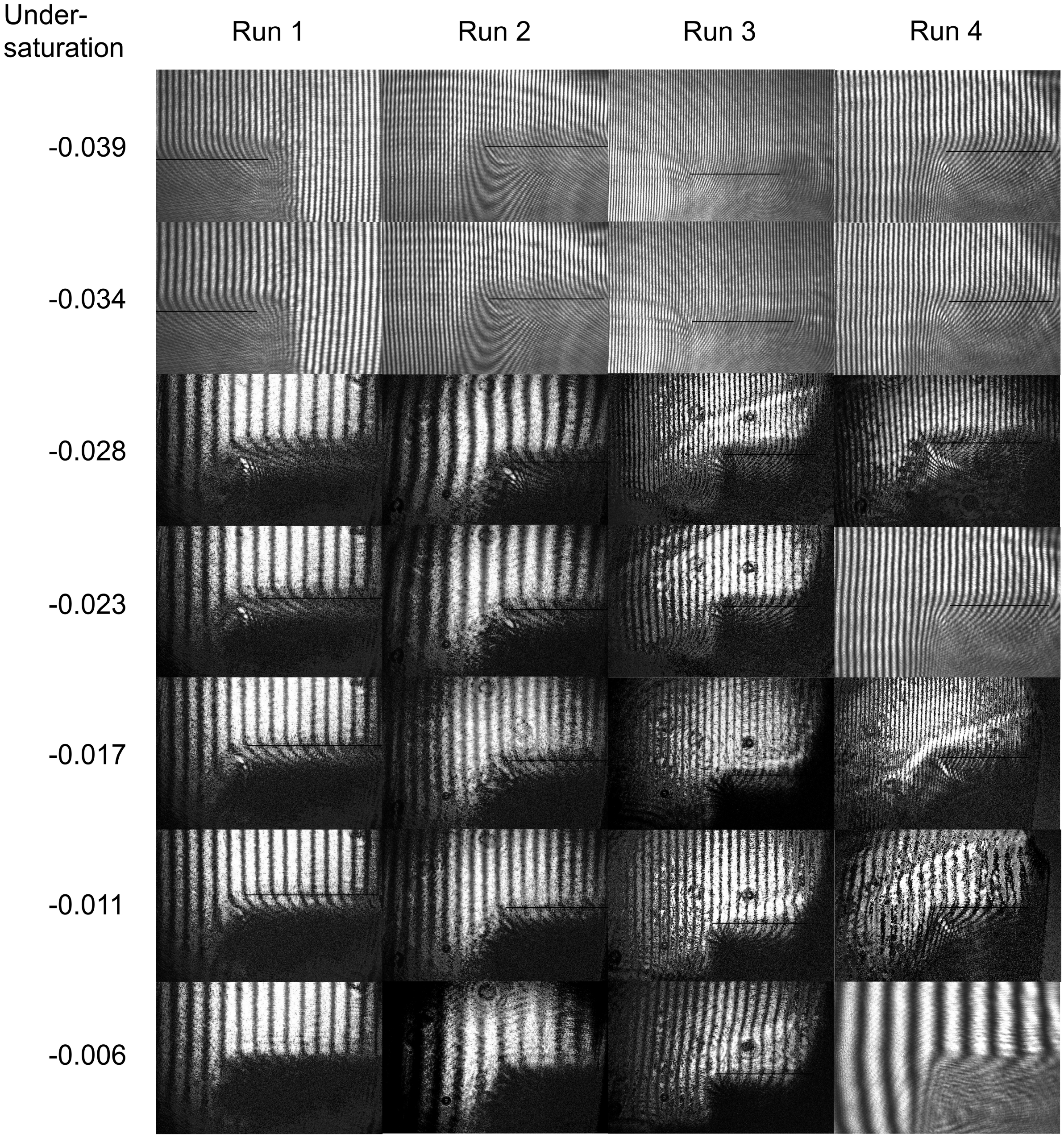

Figure S10. The 4 runs of interferograms of L-alanine dissolving in water at 23.5 ^{0}C/-0.039, 23 ^{0}C/-0.034, 22.5 ^{0}C/-0.028, 22 ^{0}C/-0.023, 21.5 ^{0}C/-0.017, 21 ^{0}C/-0.011 and 20.5 ^{0}C/-0.006. Fringes were aligned perpendicular to the {120} crystal surface and all images have the same scale as in Run 1 0.029 in Figure S9.

References

1) Hooper, R. M.; McArdle, B. J.; Narang, R. S.; Sherwood, J. N. Crystallization from solution at low temperatures. In *Crystal Growth,* 2nd ed.; Pamplin, B. R., Eds.; Oxford, UK: Pergamon, **1980**, 16, 395-420.

2) Lehmann, M. S.; Koetzle, T. F.; Hamilton, W. C. Precision Neutron Diffraction Structure Determination of Protein and Nucleic Acid Components. I. The Crystal and Molecular Structure of the Amino Acid L-Alanine. *Journal of the American Chemical Society.* **1972**, 94(8), 2657-2660.

3) Bruno, I. J.; Cole, J. C.; Edgington, P.R.; Kessler, M.; Macrae, C. F.; McCabe, P.; Pearson, J.; Taylor, R. New software for searching the Cambridge Structural Database and visualizing crystal structures. *Acta Crystallographica Section B: Structural Science.* **2002**, 58(3), 389-397.

4) *Mopac Quantum Chemistry Program Exchange Program No. 455*, v6.0; Indiana University, Bloomington, IN.

5) Momany, F. A.; Carruthers, L. M.; McGuire, R. F.; Scheraga, H. A. Intermolecular potentials from crystal data. III. Determination of empirical potentials and application to the packing configurations and lattice energies in crystals of hydrocarbons, carboxylic acids, amines, and amides. *The Journal of Physical Chemistry.* **1974**, 78(16), 1595-1620.

6) Clydesdale, G.; Docherty, R.; Roberts, K. J. HABIT - a program for predicting the morphology of molecular crystals. *Computer Physics Communications.* **1991**, 64(2), 311-328.

7) Razzetti, C.; Ardoino, M.; Zanotti, L.; Zha, M.; Paorici, C. Solution growth and characterisation of L-alanine single crystals. *Crystal Research and Technology.* **2002**, 37(5), 456-465.

8) Lechuga-Ballesteros, D.; Rodríguez-Hornedo, N. Growth and morphology of L-alanine crystals: influence of additive adsorption. *Pharmaceutical research.* **1993**, 10(7), 1008-1014.

9) Lechuga-Ballesteros, D.; Rodriguez-Hornedo, N. Effects of molecular structure and growth kinetics on the morphology of L-alanine crystals. *International Journal of Pharmaceutics.* **1995**, 115(2), 151-160.

10) Clydesdale, G.; Docherty, R.; Roberts, K. J. A predictive approach to modelling the morphology of organic crystals based on crystal structure using the atom-atom method. In *Crystal Growth: Proceedings of the Crystal Growth – 3rd European Conference*. Budapest, Hungary, May 5-11, **1991**; Lörinczy, A., Eds.; TransTech Publications: Zurich, Switzerland, 1991, Crystal Properties and Preparation 36-38, 234-243.

11) Donnay, J. D. H.; Harker, D. A new law of crystal morphology extending the law of Bravais. *American Mineralogist: Journal of Earth and Planetary Materials.* **1937**, 22(5), 446-467.

12) Raghavalu, T.; Ramesh Kumar, G.; Gokul Raj, S.; Mathivanan, R.; Mohan, R. Nucleation thermodynamical studies on nonlinear optical L-alanine single crystals. *Journal of Crystal Growth.* **2007**, 307(1), 112-115.

13) Durga, K. K. H.; Selvarajan, P.; Shanthi, D. Nucleation Kinetics, XRD and SHG Studies of L-Alanine Single Crystals Grown at Different Supersaturation Levels. *International Journal of Current Research and Review.* **2012**, 4(14), 68-77.

14) Bowden, N. A. Modelling the Solubility of the 20 Proteinogenic Amino Acids with Experimentally Derived Saturation Data. Ph.D. Dissertation, Wageningen, New Zealand: University of Wageningen, **2018**.

15) An, M.; Qiu, J.; Yi, D.; Liu, H.; Hu, S.; Han, J.; Huang, H.; He, H.; Liu, C.; Zhao, Z.; Shi, Y.; Wang, P. Measurement and Correlation for Solubility of L-Alanine in Pure and Binary Solvents at Temperatures from 283.15 to 323.15 K. *Journal of Chemical & Engineering Data.* **2020**, 65(2), 549-560.

16) Needham, T. E. The Solubility of Amino Acids in Various Solvent Systems The Solubility of Amino Acids in Various Solvent Systems. Ph.D. Dissertation, Kingston, Rhode Island: University of Rhode Island, **1970**.

17) Gekko, K. Mechanism of polyol-induced protein stabilization: solubility of amino acids and diglycine in aqueous polyol solutions. *The Journal of Biochemistry.* **1981**, 90(6), 1633-1641.

18) Orella, C. J.; Kirwan, D. J. Correlation of Amino Acid Solubilities in Aqueous Aliphatic Alcohol Solutions. *Industrial and Engineering Chemical Research.* **1991**, 30(5), 1040-1045.

19) Jin, X. Z.; Chao, K. C. Solubility of Four Amino Acids in Water and of Four Pairs of Amino Acids in Their Water Solutions. *Journal of Chemical & Engineering Data.* **1992**, 37(2), 199-203.
